# Supplementary figures and images for: YAP/TAZ deficiency reprograms macrophage phenotype and improves infarct healing and cardiac function after myocardial infarction
Source: PLoS Biol. 2020 Dec 2;18(12):e3000941. doi: 10.1371/journal.pbio.3000941 (PMC7735680; doi:10.1371/journal.pbio.3000941)

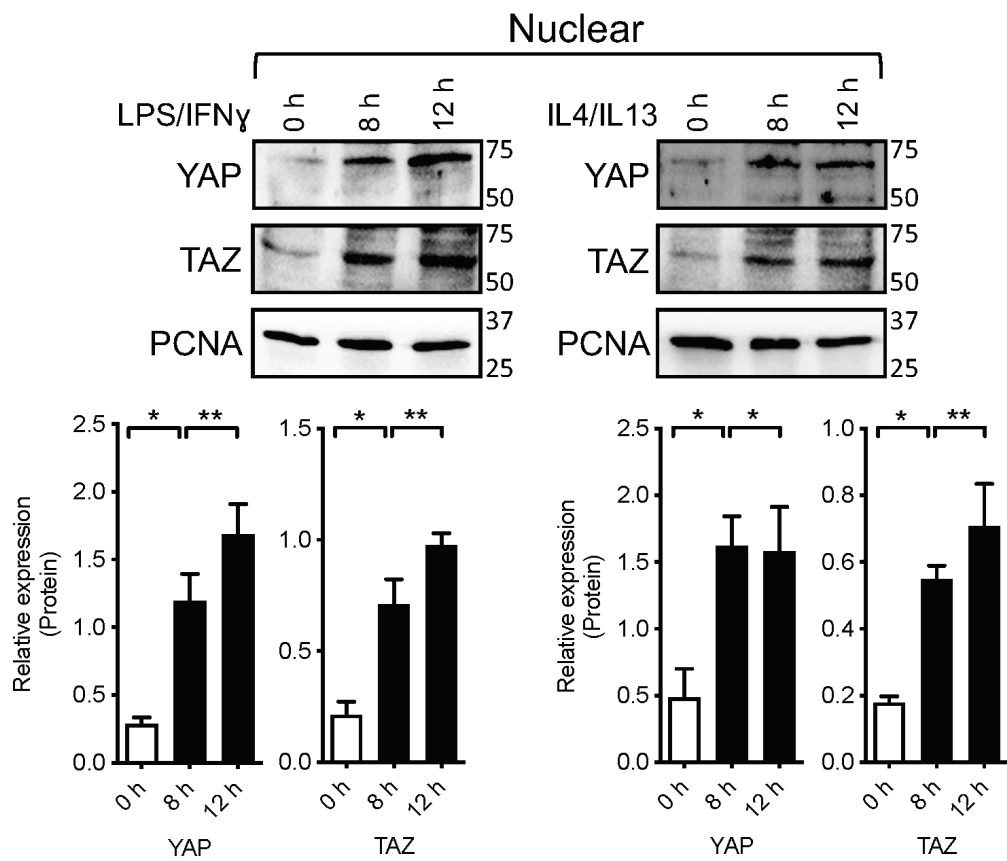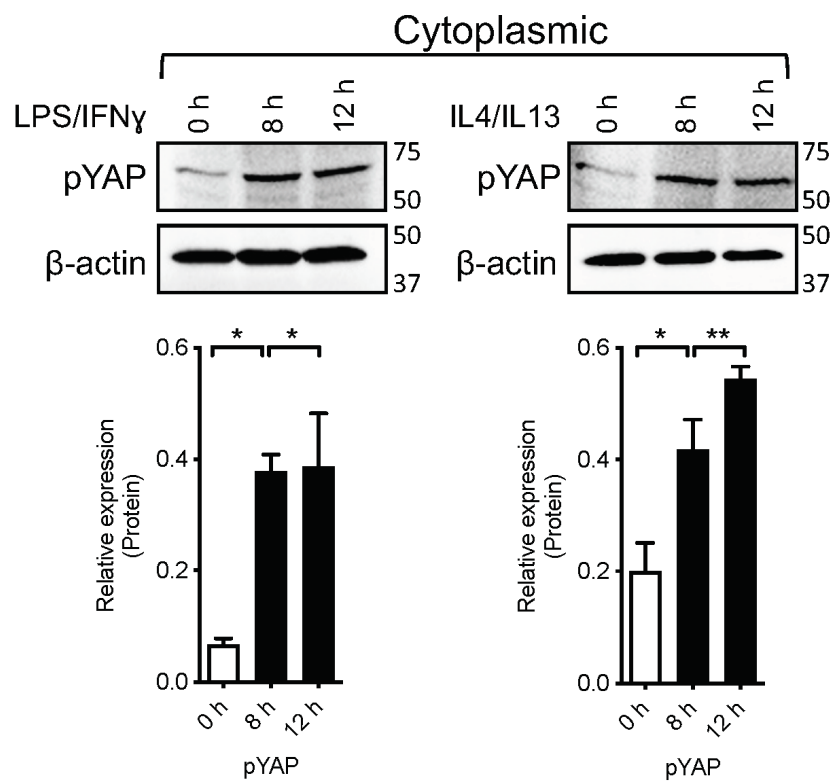

Supplement: S1 Fig — BMDMs were isolated from wild-type mice and stimulated with/without LPS/IFNγ or IL4/IL13 for 0, 8 and 12 hours, respectively. Western blot analysis for YAP and TAZ was performed using the nuclear fraction of BMDMs. PCNA is shown as a loading control. The relative expression was quantified. Western blot analysis for pYAP was performed using the cytoplasmic fraction of BMDMs. Β-actin is shown as a loading control. The relative expression was quantified. For numerical raw data, please see S1 Data. (PDF) [file pbio.3000941.s001.pdf]

**A**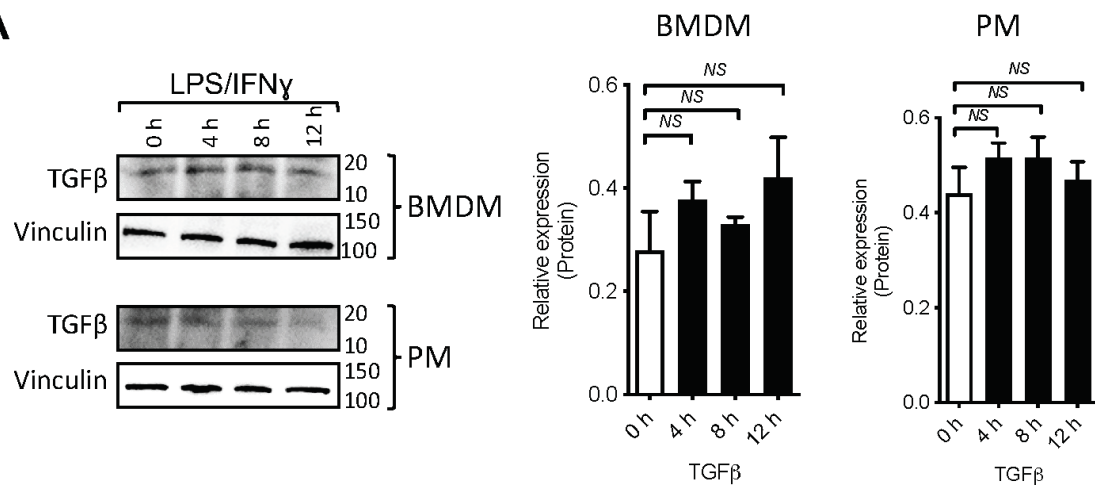**B**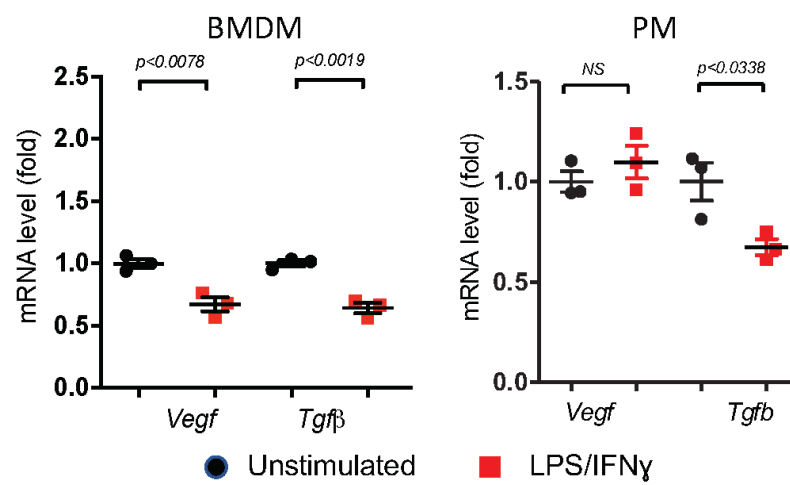**C**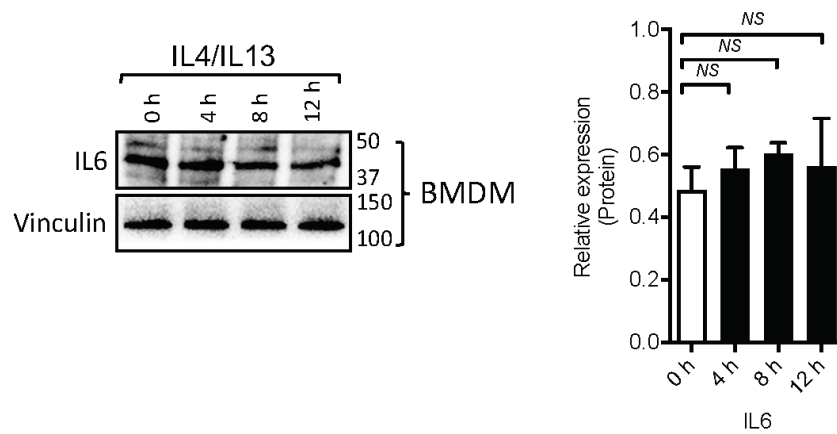

Supplement: S2 Fig — (A) BMDMs and PMs were isolated from wild-type mice and stimulated with/without LPS/IFNγ for 0, 4, 8, and 12 hours, respectively. Western blot analysis for TGFβ using cell lysates of BMDMs and PMs. Vinculin is shown as a loading control. The relative expression was quantified. (B) BMDMs and PMs were isolated from wild-type mice and stimulated with/without LPS/IFNγ for 12 hours. Real-time qPCR for reparative marker genes, Vegf and Tgfβ, using RNA isolated from untreated or LPS/IFNγ-treated macrophages. (C) BMDMs were isolated from wild-type mice and stimulated with/without IL4/IL13 for 0, 4, 8, and 12 hours, respectively. Western blot analysis for IL6 using cell lysates of BMDMs. Vinculin is shown as a loading control. The relative expression was quantified. For numerical raw data, please see S1 Data. (PDF) [file pbio.3000941.s002.pdf]

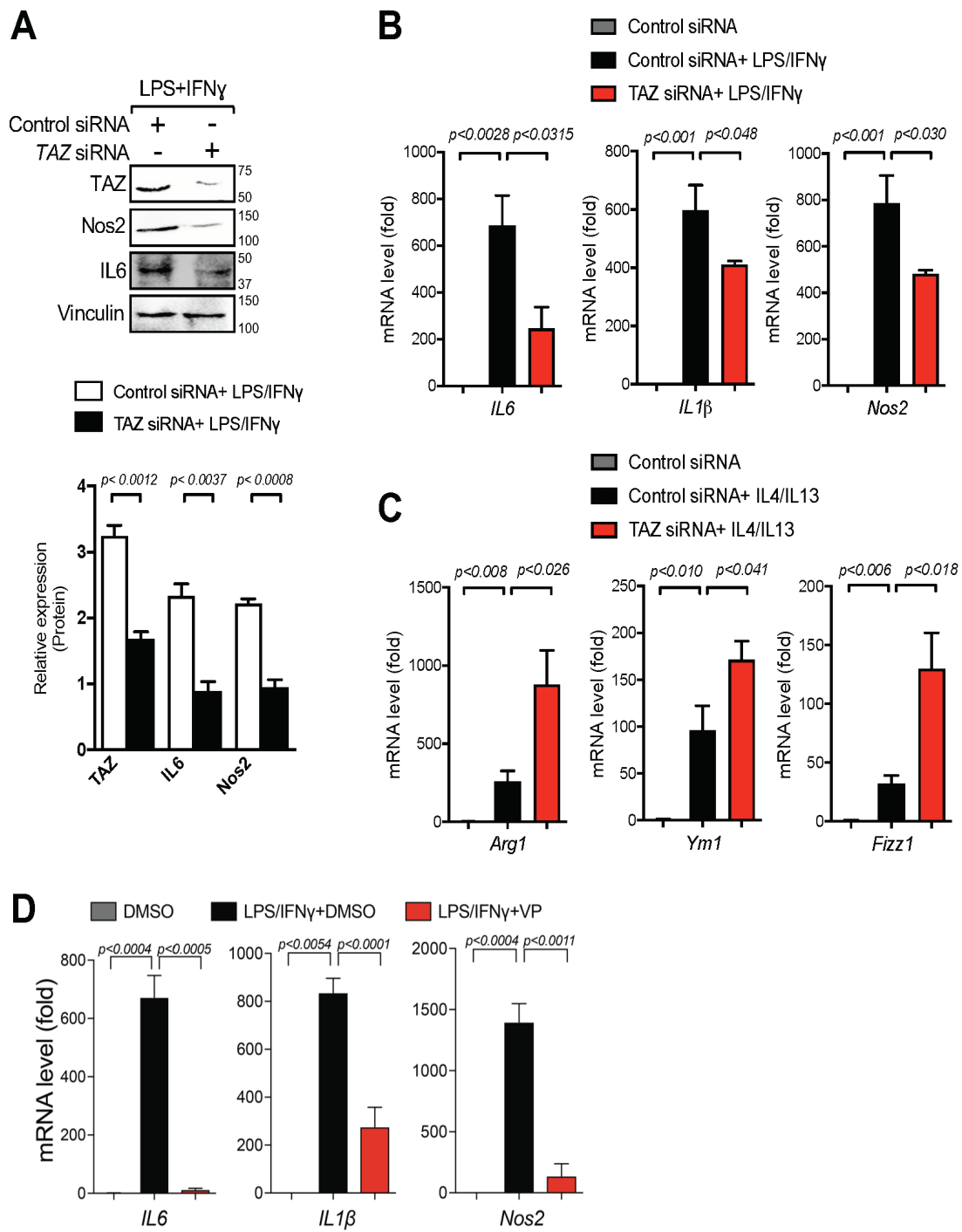

Supplement: S3 Fig — (A–C) BMDMs were isolated from wild-type mice and transfected with control or TAZ siRNA for 72 hours, followed by LPS/IFNγ or IL4/IL13 stimulation for 16 hours. Cell lysates were prepared for western blot and qRT-PCR analysis. (A) Western blot analysis for TAZ, IL6, and Nos2 was performed using total lysates from wild-type BMDMs transfected with control or TAZ siRNA. Vinculin is shown as a loading control. The relative expression was quantified. (B) Real-time qPCR for pro-inflammatory marker genes IL6, IL1β, and Nos2 using RNA isolated from wild-type BMDMs transfected with control or TAZ siRNA and stimulated with LPS/IFNγ. (C) Real-time qPCR for reparative marker genes Arg1, Ym1, and Fizz1 using RNA isolated from wild-type BMDMs transfected with control or TAZ siRNA and stimulated with IL4/IL13. (D) Real-time qPCR for IL6, IL1β, and Nos2 using RNA isolated from BMDMs treated with either DMSO, LPS/IFNγ, or LPS/IFNγ together with verteporfin (VP). Data are shown as the mean ± SEM, n = 3 for each experimental group. Gene expression data were normalized with the reference gene Gapdh, and results are represented as fold change relative to the control treatment. For numerical raw data, please see S1 Data. (PDF) [file pbio.3000941.s003.pdf]

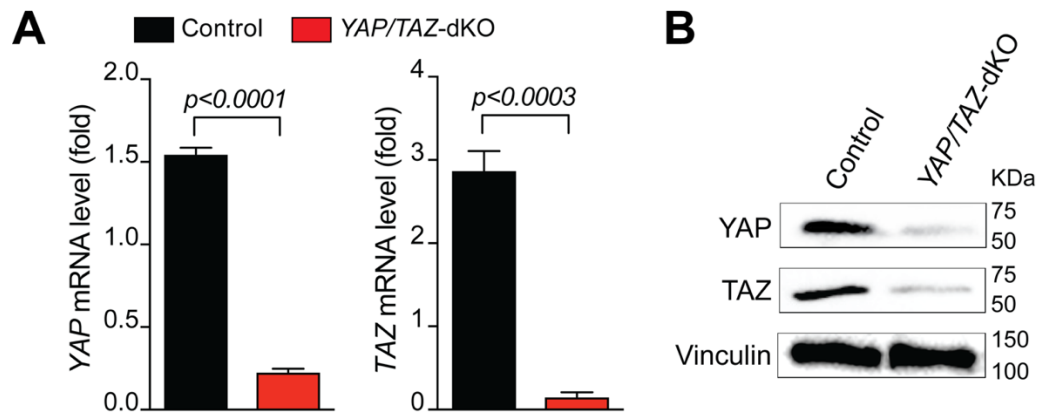

Supplement: S4 Fig — (A) Real-time qPCR for YAP and TAZ using RNA isolated from control and YAP/TAZ-dKO BMDMs. n = 3 in each group. (B) Western blot analysis for YAP and TAZ was performed using total lysates from control and YAP/TAZ-dKO BMDMs. Vinculin is shown as a loading control. For numerical raw data, please see S1 Data. (PDF) [file pbio.3000941.s004.pdf]

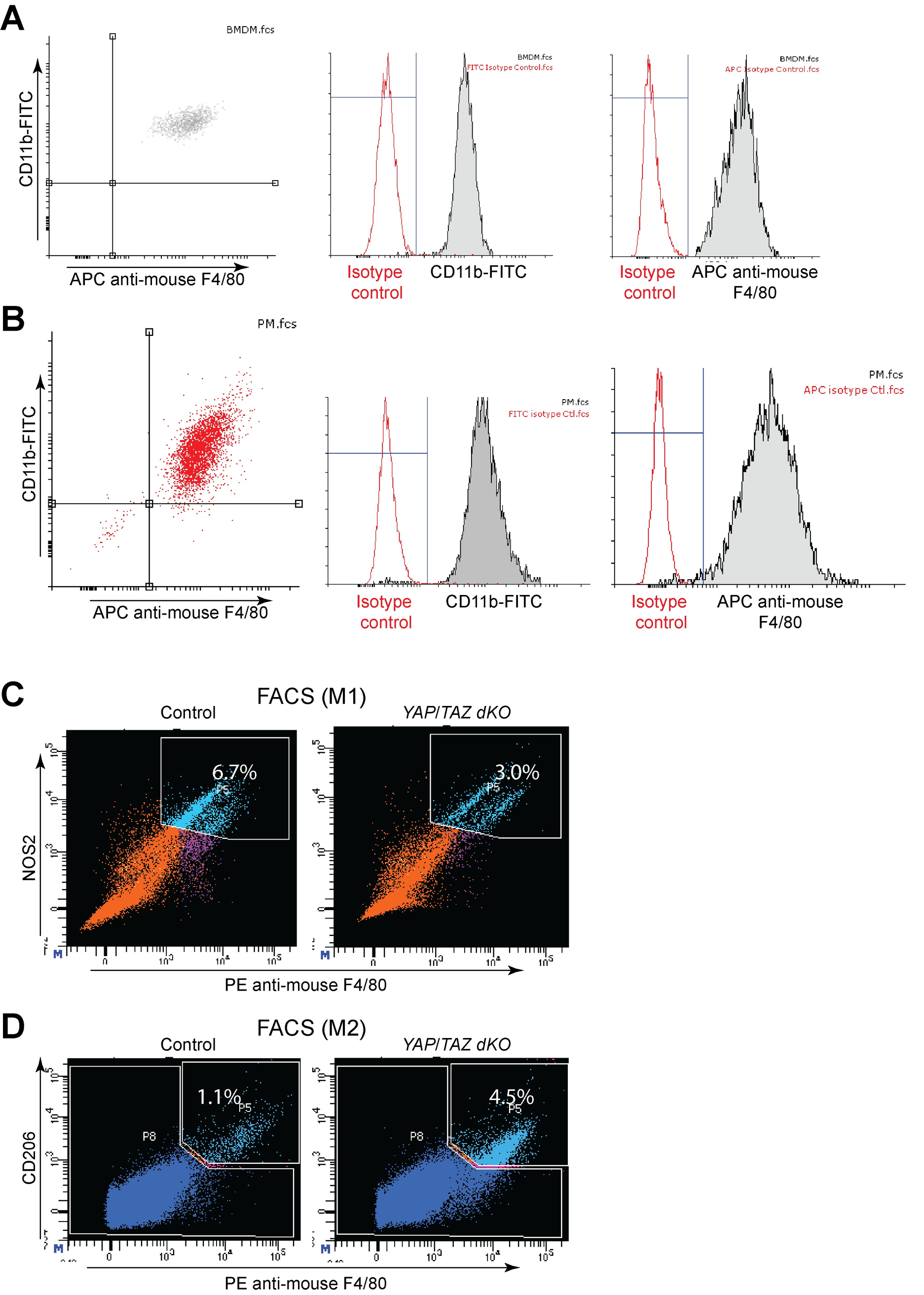

Supplement: S5 Fig — (A and B) Isolated BMDMs (A) and PMs (B) were stained with CD11b-FITC and APC anti-mouse F4/80 antibodies, and their purity were evaluated by flow cytometry analysis. (C and D) Representative flow cytometry analysis of pro-inflammatory (iNOS+/F4/80+) and reparative (CD206+/F4/80+) macrophages in total macrophages from the 2 and 6 days post-MI hearts, respectively; showing the percentage of F4/80+iNOS+ and F4/80+CD206+ macrophages in YAP/TAZ-dKO hearts compared to respective control. (TIF) [file pbio.3000941.s005.tif]

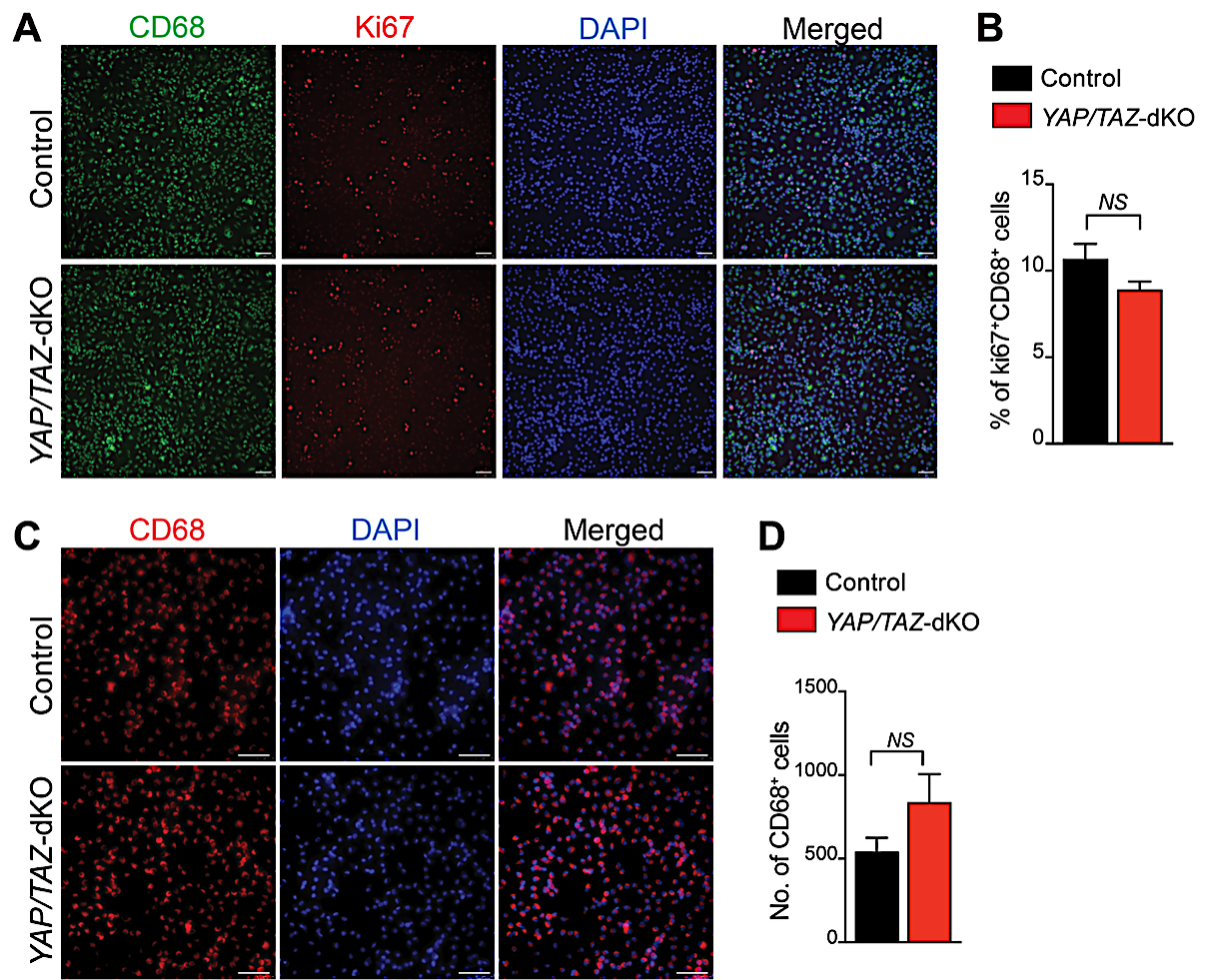

Supplement: S6 Fig — (A) To assess changes in BMDMs proliferation, cultured BMDMs were stimulated with LPS/IFNɣ and immunostained with CD68 and Ki67. DAPI was used to stain nuclei. Scale bar 100 μM. (B) Quantification of CD68 and Ki67 double-positive BMDMs from control and YAP/TAZ-dKO mice. (C) Migration of control and YAP/TAZ-dKO BMDMs was examined by a transwell assay. Cells that had migrated to the lower chamber of the transwell plate were visualized by CD68 immunostaining. DAPI was used to stain nuclei. Scale bar 50 μM. (D) Quantification of CD68 positive BMDMs from control and YAP/TAZ-dKO mice migrated to the lower chamber of the transwell plate. For numerical raw data, please see S1 Data. (PDF) [file pbio.3000941.s006.pdf]

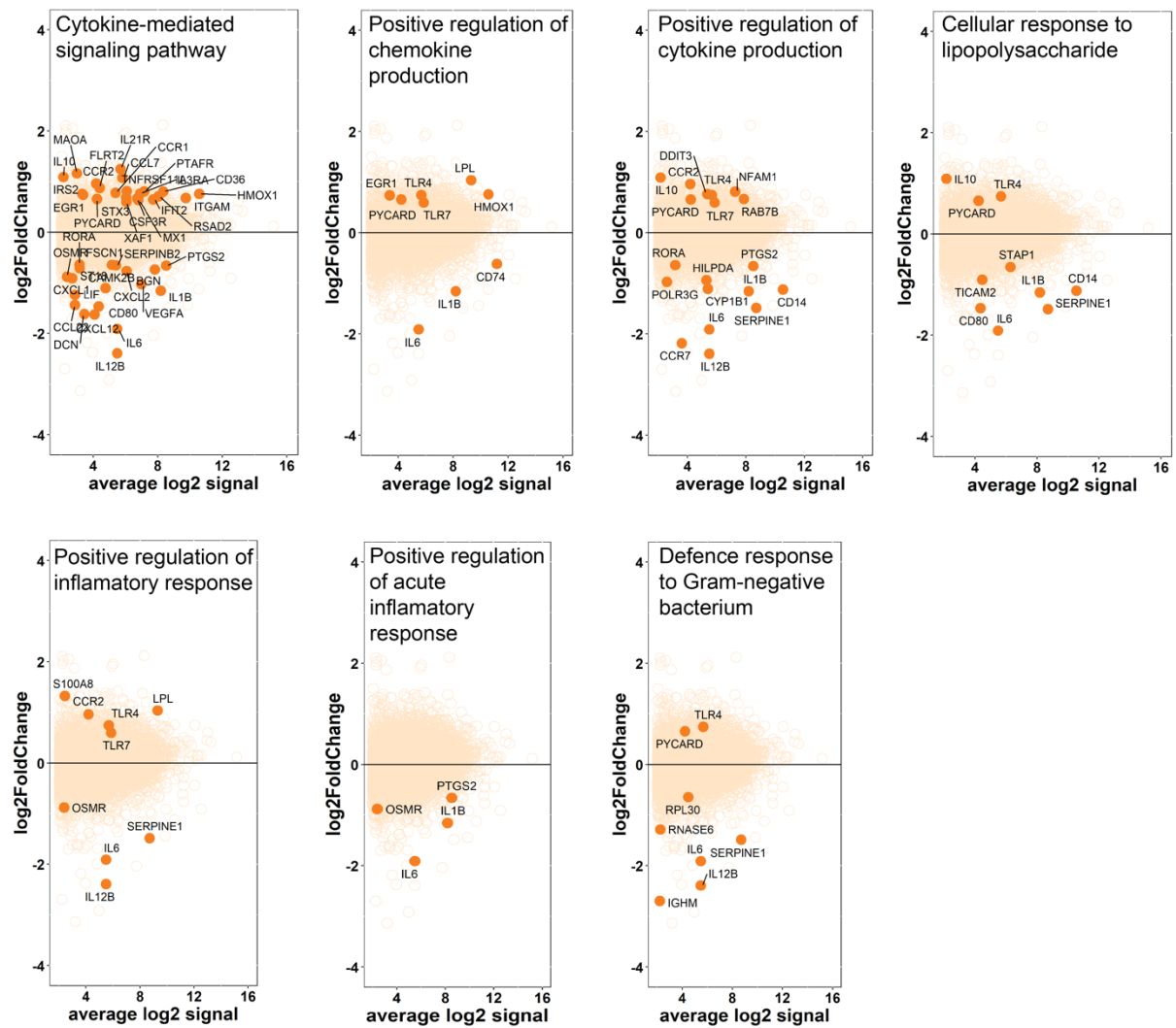

Supplement: S7 Fig — MA plots for pathways identified from pathway enrichment analysis of the RNA-seq data from untreated or LPS/IFNγ treated control and YAP/TAZ-dKO BMDMs. For numerical raw data, please see S1 Data. (PDF) [file pbio.3000941.s007.pdf]

**A**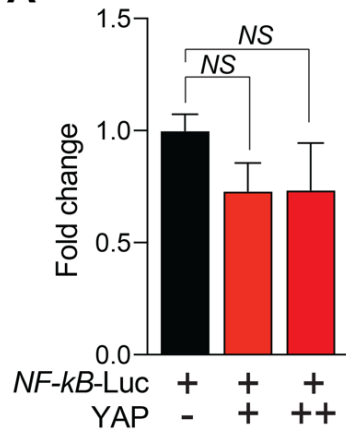**B**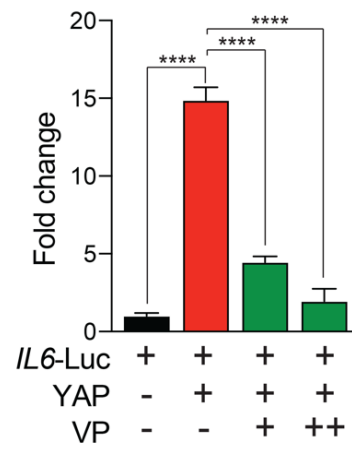**C**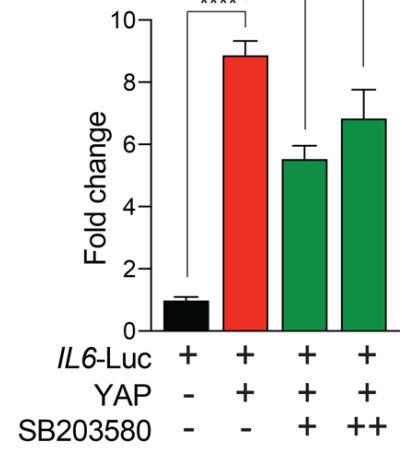

Supplement: S8 Fig — Two luciferase reporters (NF-kB-Luc and IL6-Luc) were used with YAP in the presence or absence of Hippo signaling inhibitor verteporfin (VP) or MAPK inhibitor SB203580. (A) Results of normalized luciferase reporter assays in HEK293T cells with NF-кB-luciferase reporter in the presence of YAP. (B) IL6-luciferase reporters were transfected in HEK293T cells with or without YAP in the presence or absence of VP or SB203580. All the experiments repeated at least 3 times. For numerical raw data, please see S1 Data. (PDF) [file pbio.3000941.s008.pdf]

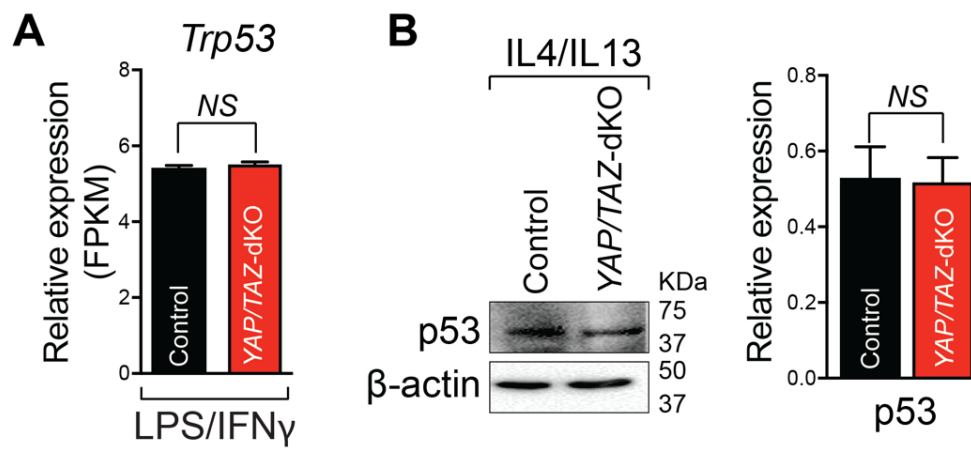

Supplement: S10 Fig — (A) Relative expression (FPKM) of Trp53 from RNA-seq analysis on control and YAP/TAZ-dKO BMDMs treated with LPS/IFNɣ. (B and C) Western blot analysis and quantification for p53 were performed using total lysates from control and YAP/TAZ-dKO BMDMs stimulated with IL4/IL13 for 24 hours. β-actin is shown as a loading control. For numerical raw data, please see S1 Data. (PDF) [file pbio.3000941.s010.pdf]

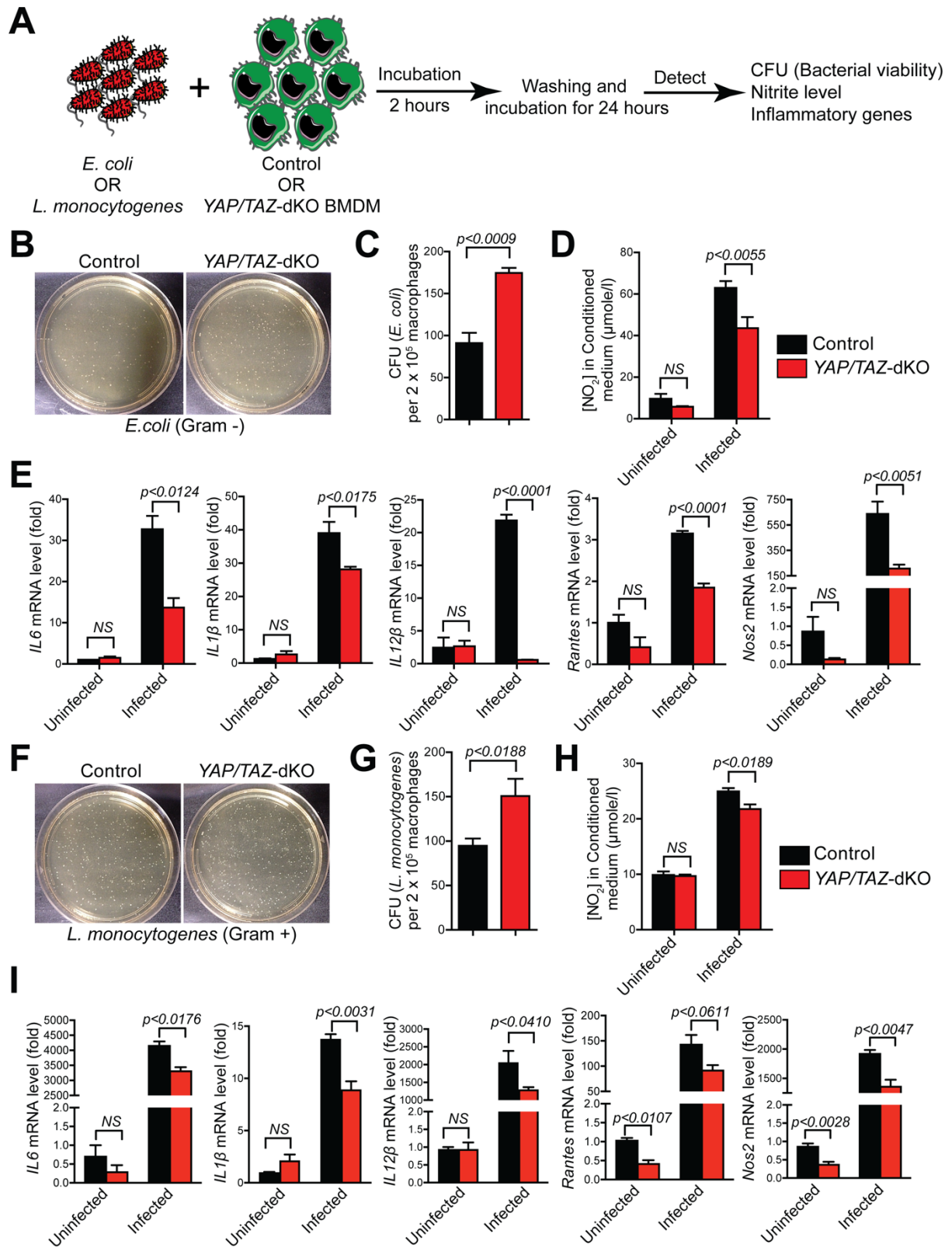

Supplement: S12 Fig — (A) Experimental design of the bacterial killing assay. Briefly, BMDMs were infected with gram-negative (E.coli) or gram-positive (L. monocytogenes) bacteria for 2 hours and after thoroughly washed, incubated for 24 hours to determine the bactericidal activity of BMDMs derived from control mice against YAP/TAZ-deficient mice. (B and C) YAP/TAZ-deficient BMDMs show reduced bactericidal activity against E. coli ex vivo. (D) Nitric oxide (NO) production determined by nitrite (NO2−) levels in conditioned medium of uninfected or E.coli infected control and YAP/TAZ-deficient BMDMs. (E) The expression of pro-inflammatory genes involved in bactericidal activity was decreased in YAP/TAZ-deficient BMDMs after incubation with E. coli. n = 3 in each group. (F and G) YAP/TAZ-deficient BMDMs exhibit reduced bactericidal activity against L. monocytogenes ex vivo. (H) Nitrite (NO2−) levels in conditioned medium of uninfected or L. monocytogenes infected control and YAP/TAZ-deficient BMDMs. (I) The expression of pro-inflammatory genes involved in bactericidal activity was decreased in YAP/TAZ-deficient BMDMs after incubation with L. monocytogenes. For numerical raw data, please see S1 Data. (PDF) [file pbio.3000941.s012.pdf]

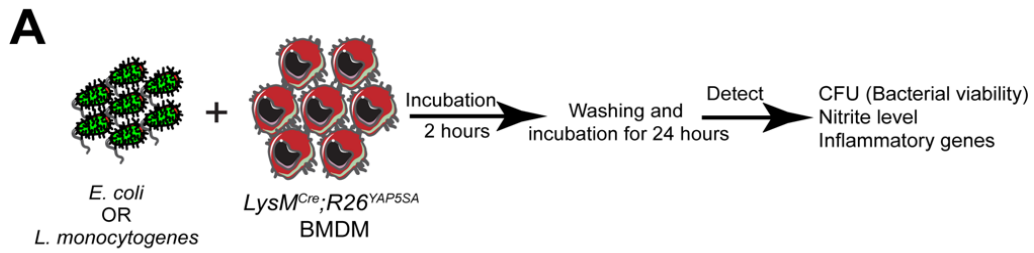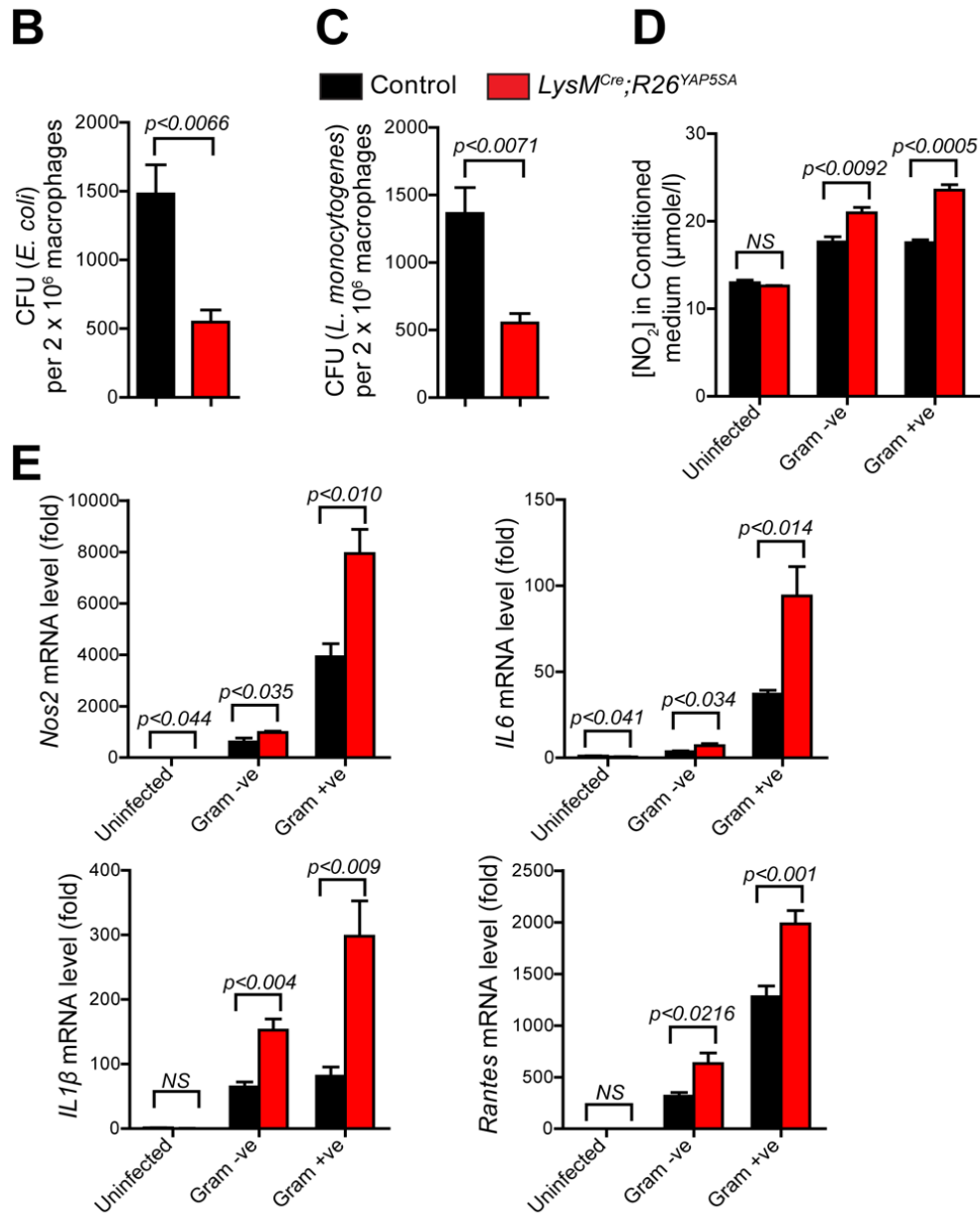

Supplement: S13 Fig — (A) Experimental design of the bacterial killing assay. Briefly, BMDMs were infected with gram-negative (E.coli) or gram-positive (L. monocytogenes) bacteria for 2 hours and after thoroughly washed, incubated for 24 hours to determine the bactericidal activity of BMDMs derived from control and YAP5SA mice. (B and C) YAP5SA BMDMs show increased bactericidal activity against E. coli and L. monocytogenes ex vivo, respectively. (D) NO production by BMDMs was determined by nitrite (NO2−) levels in conditioned medium. (E) The expression of pro-inflammatory genes involved in bactericidal activity was enhanced in YAP5SA BMDMs after incubation with E. coli or L. monocytogenes. n = 3 in each group. For numerical raw data, please see S1 Data. (PDF) [file pbio.3000941.s013.pdf]

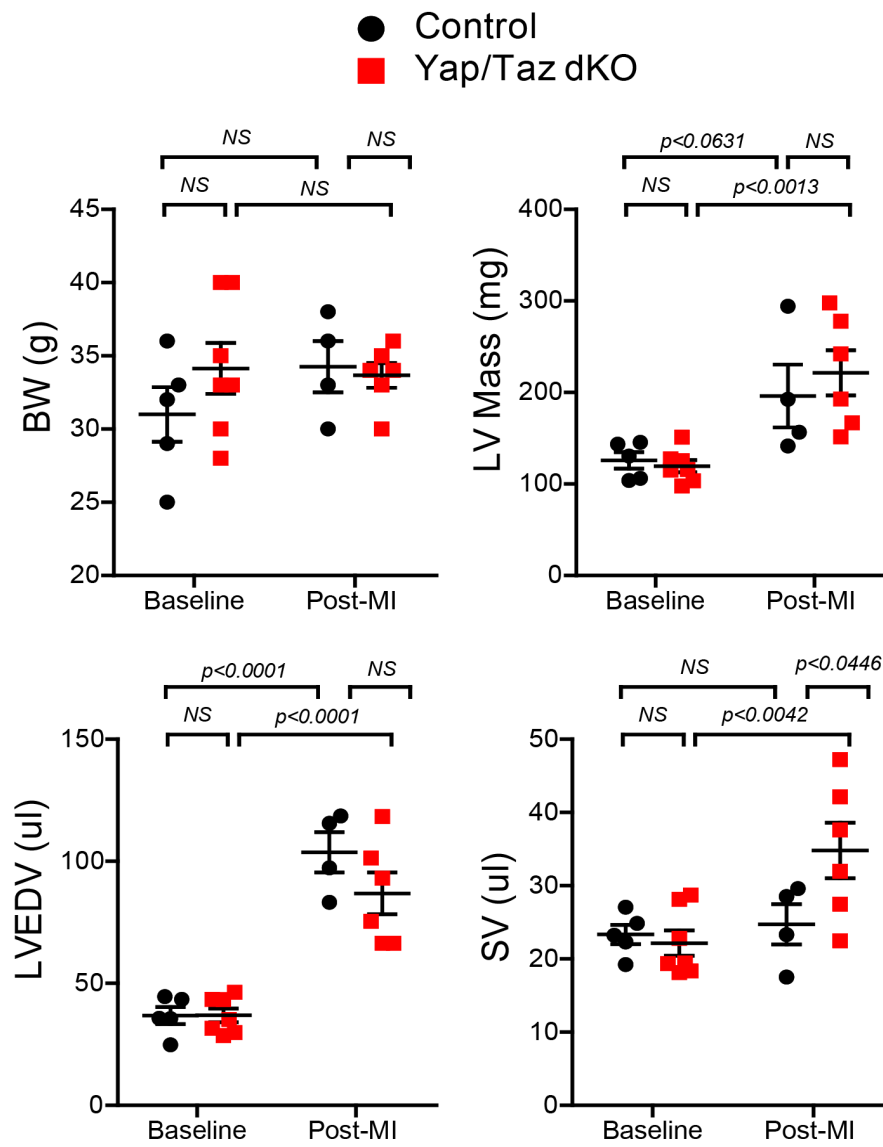

Supplement: S14 Fig — Echocardiographic measurements of ventricular functional parameters at baseline and 4 weeks post-MI from control and YAP/TAZ-dKO mice: body weight (BW); and left ventricular mass (LV Mass); left ventricular end-diastolic volume (LVEDV); and stroke volume (SV). Data are shown as mean ± SEM (n = 4 to 7 per group). For numerical raw data, please see S1 Data. (PDF) [file pbio.3000941.s014.pdf]

**A**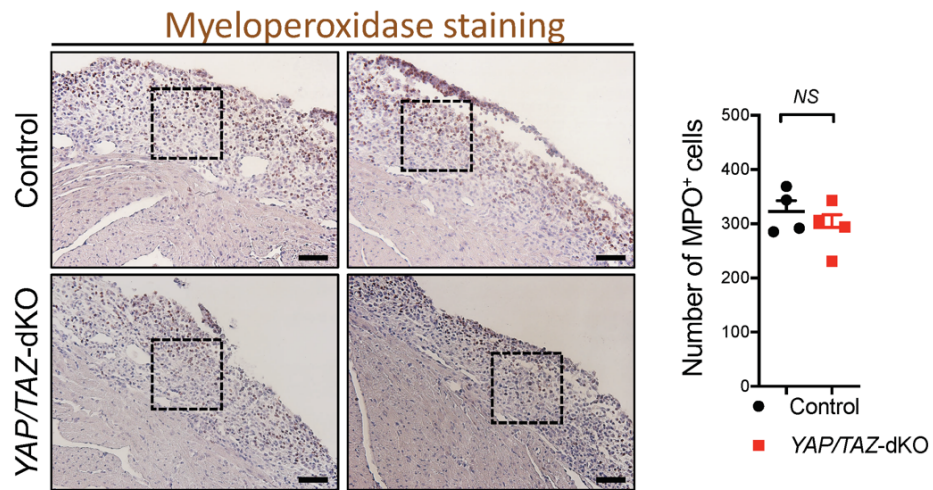**B**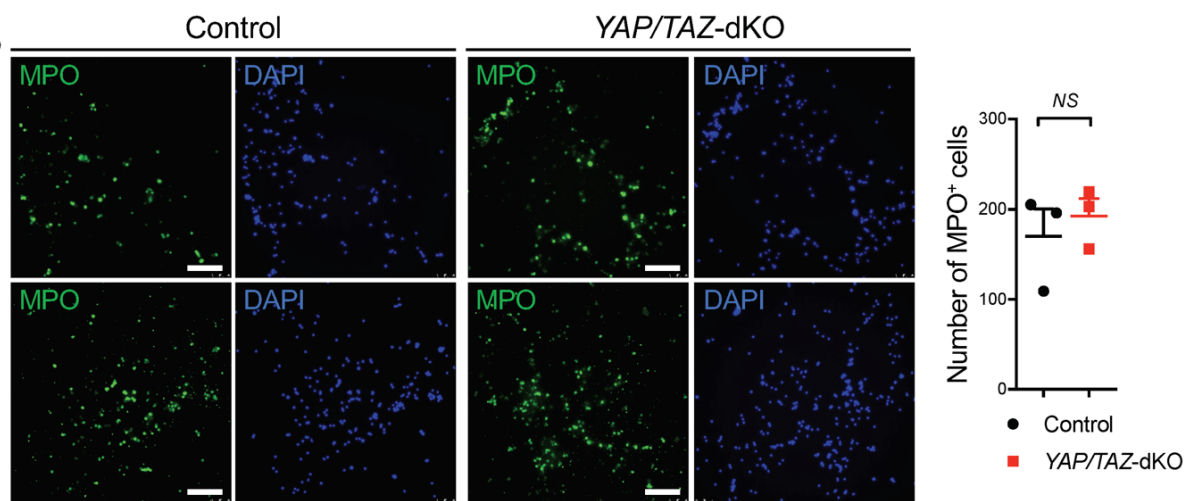

Supplement: S15 Fig — (A) Immunohistochemistry and quantification for MPO on control and YAP/TAZ-dKO infarcted heart sections at 2 days post-MI. The number of MPO positive cells was determined in ≥5 distinct microscope fields for each heart section. At least 4 hearts were analyzed for each group. Scale bar represents 100 μm. (B) Migration of control and YAP/TAZ-dKO neutrophils was examined by a transwell assay. Cells that had migrated to the lower chamber of the transwell plate were visualized by MPO immunostaining. DAPI was used to stain nuclei. Scale bar 100 μM. Quantification of MPO positive neutrophils from control and YAP/TAZ-dKO mice migrated to the lower chamber of the transwell plate. For numerical raw data, please see S1 Data. (PDF) [file pbio.3000941.s015.pdf]

**A**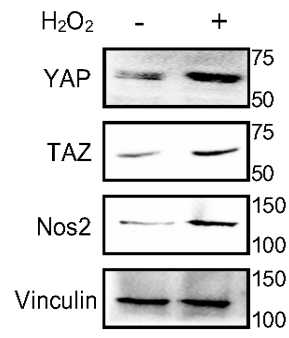**B**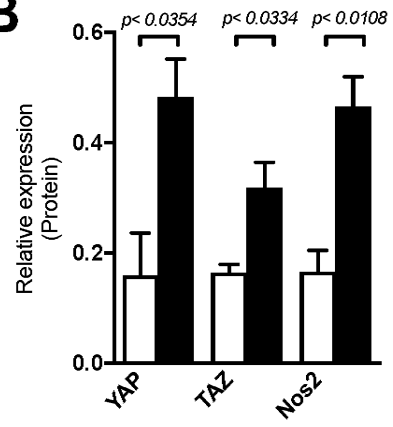

Supplement: S16 Fig — (A) BMDMs were isolated from wild-type mice and exposed to hypoxia with H2O2 (100 μM) for 8 hours. Western blot analysis for YAP, TAZ, and Nos2 was performed using total lysates from unstimulated and H2O2-stimulated cells. Vinculin is shown as a loading control. (B) The relative protein expression of YAP, TAZ, and Nos2 was quantified. For numerical raw data, please see S1 Data. (PDF) [file pbio.3000941.s016.pdf]

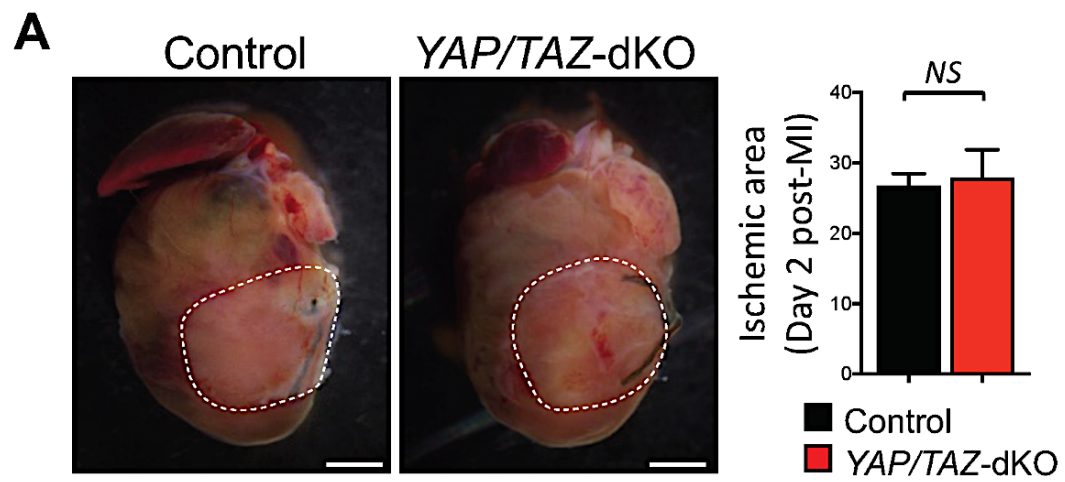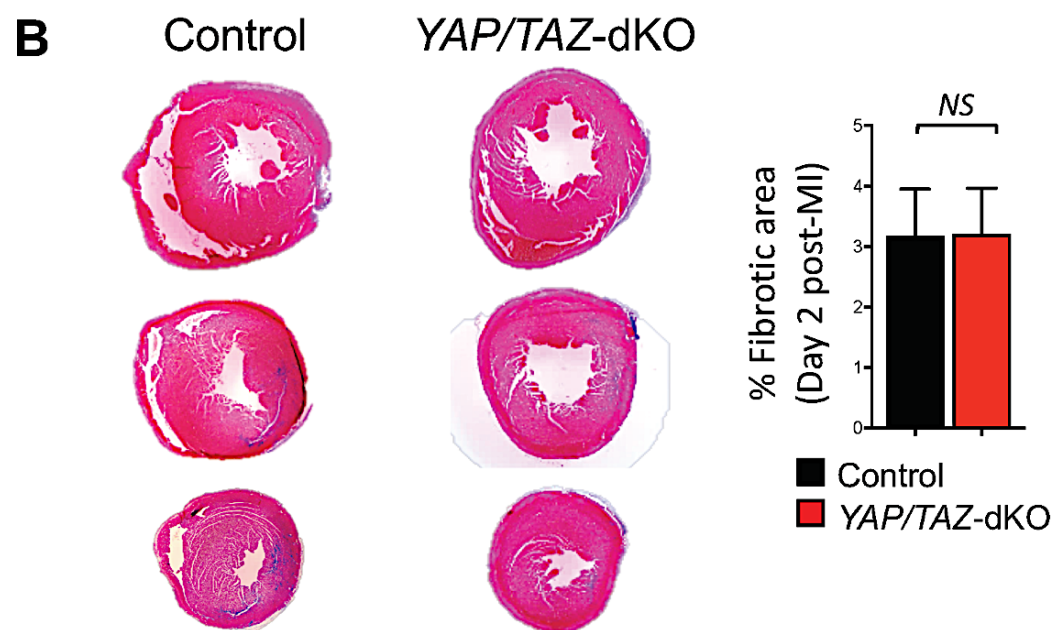

Supplement: S17 Fig — (A) Whole-mount images of control and mutant animals as indicated, 2 days post-MI. The white dotted line indicates the area of injury near the ligature. Scale bar 500 μM. Quantification of Ischemic area, (n = 3 per group). (B) Masson's trichrome staining and quantification of fibrosis on control and YAP/TAZ-dKO heart at 2 days post-MI. The area was measured in 5≥ distinct microscope field, and the fibrotic area was normalized to the remaining heart for each heart section. At least 3 hearts were analyzed for each group. For numerical raw data, please see S1 Data. (PDF) [file pbio.3000941.s017.pdf]

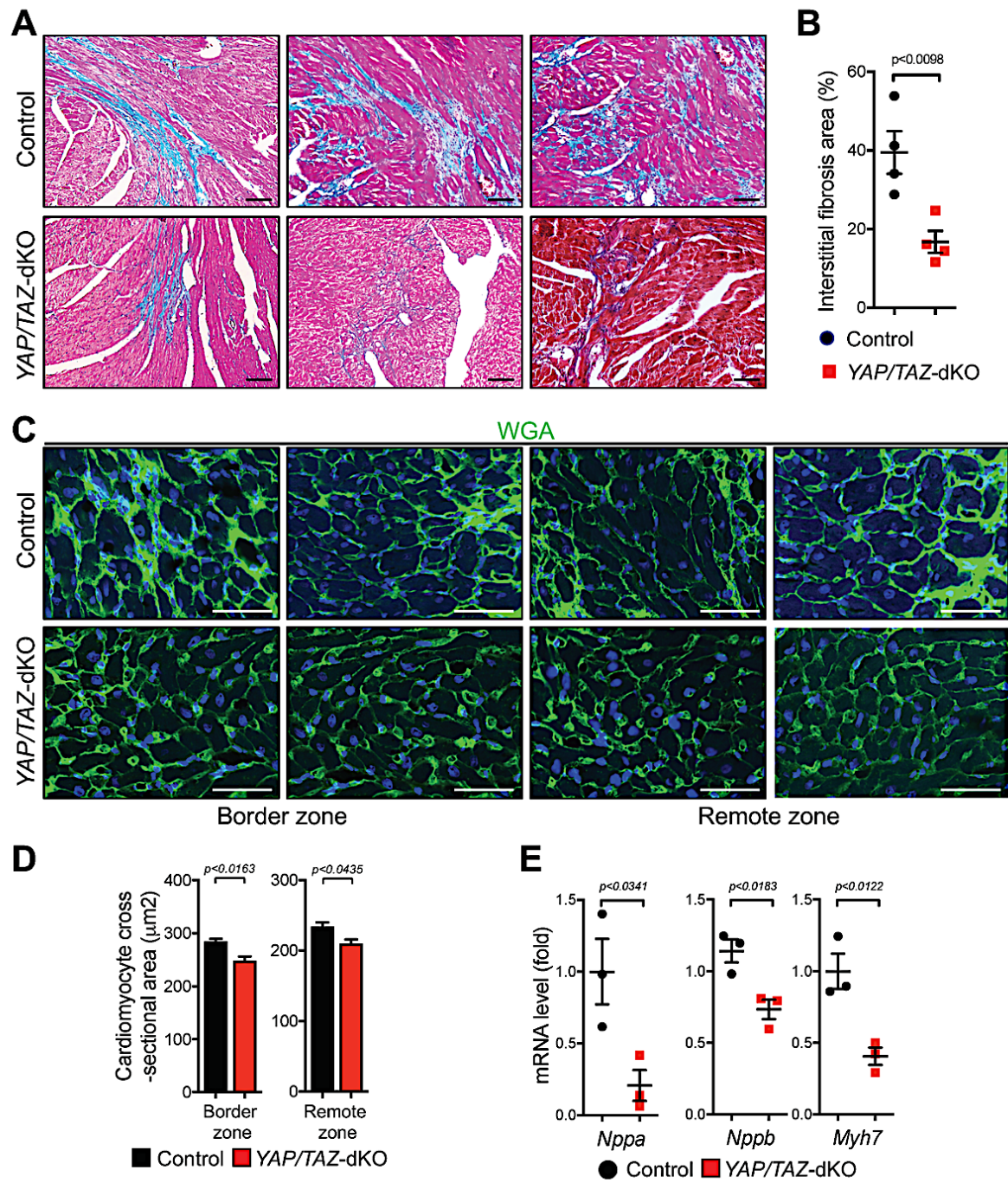

Supplement: S18 Fig — (A and B) Masson's trichrome staining and quantification of interstitial myocardial fibrosis on control and YAP/TAZ-dKO heart sections at 28 days post-MI. The area was measured in 5≥ distinct microscope field, and the fibrotic area was normalized to the remaining heart for each heart section. At least 4 hearts were analyzed for each group. Scale bar represents 100 μm. (C and D) The cross-sectional area of the infarcted heart (border and remote zone) from control and YAP/TAZ dKO mice at 28 days post-MI. The area was measured in ≥5 distinct microscope fields for each heart section. At least 3 hearts were analyzed for each group. Scale bar represents 50 μm. (E) The expression of genes such as Myh7, Nppa, and Nppb involved in hypertrophy was reduced in YAP/TAZ-dKO heart compared to control at 28 days post-MI. n = 3 in each group. For numerical raw data, please see S1 Data. (PDF) [file pbio.3000941.s018.pdf]

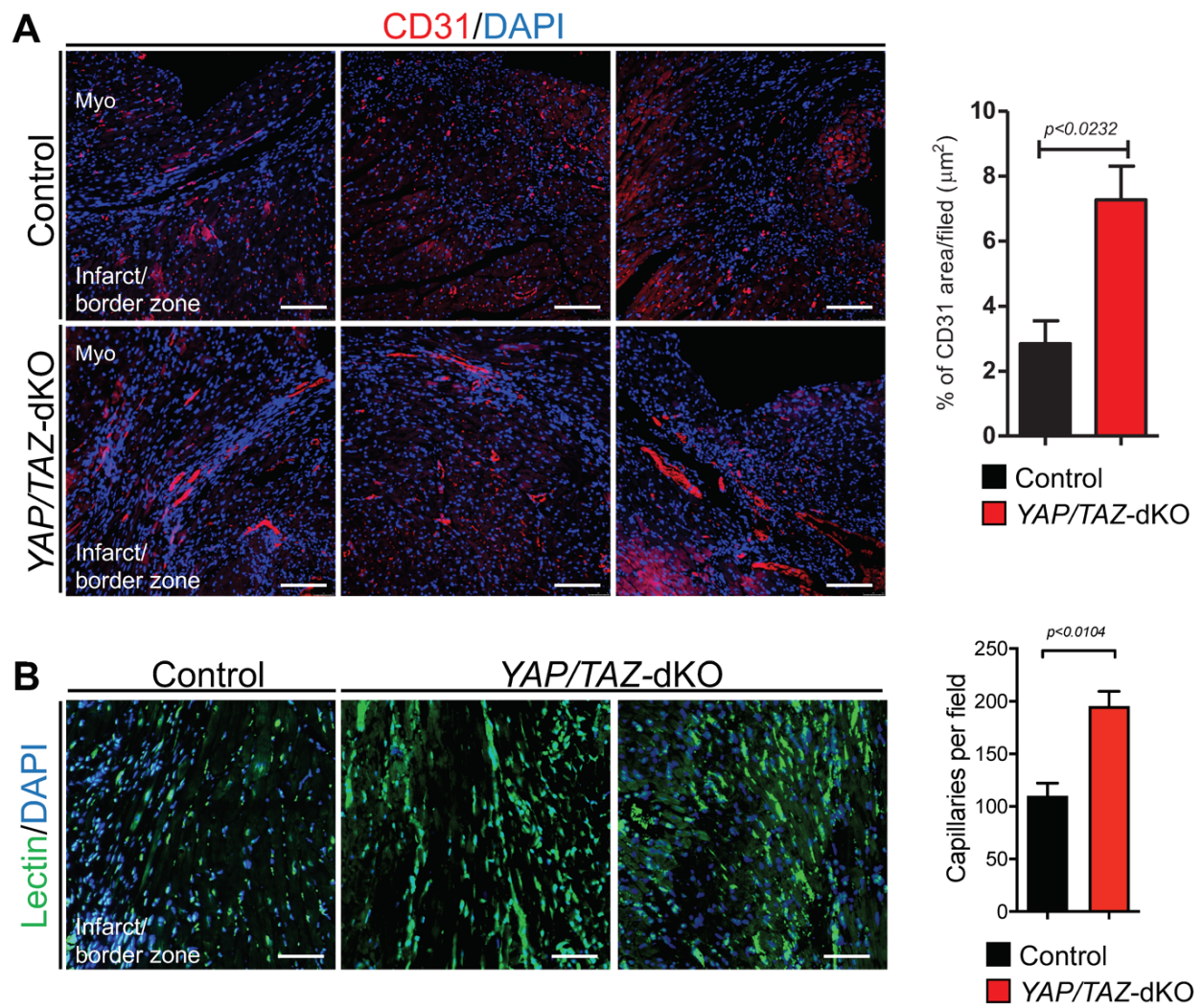

Supplement: S19 Fig — (A) Immunohistochemistry and quantification for CD31 on control and YAP/TAZ-dKO heart sections at 28 days post-MI. The area was measured in ≥3 distinct microscope fields for each heart section. At least 3 hearts were analyzed for each group. Scale bar represents 100 μm. (B) Increased capillary density in YAP/TAZ-dKO heart at 28 days post-MI along with the corresponding quantification. At least 3 hearts were analyzed for each group. Scale bar 50 μM. For numerical raw data, please see S1 Data. (PDF) [file pbio.3000941.s019.pdf]

# BD FACSDiva 9.0.1

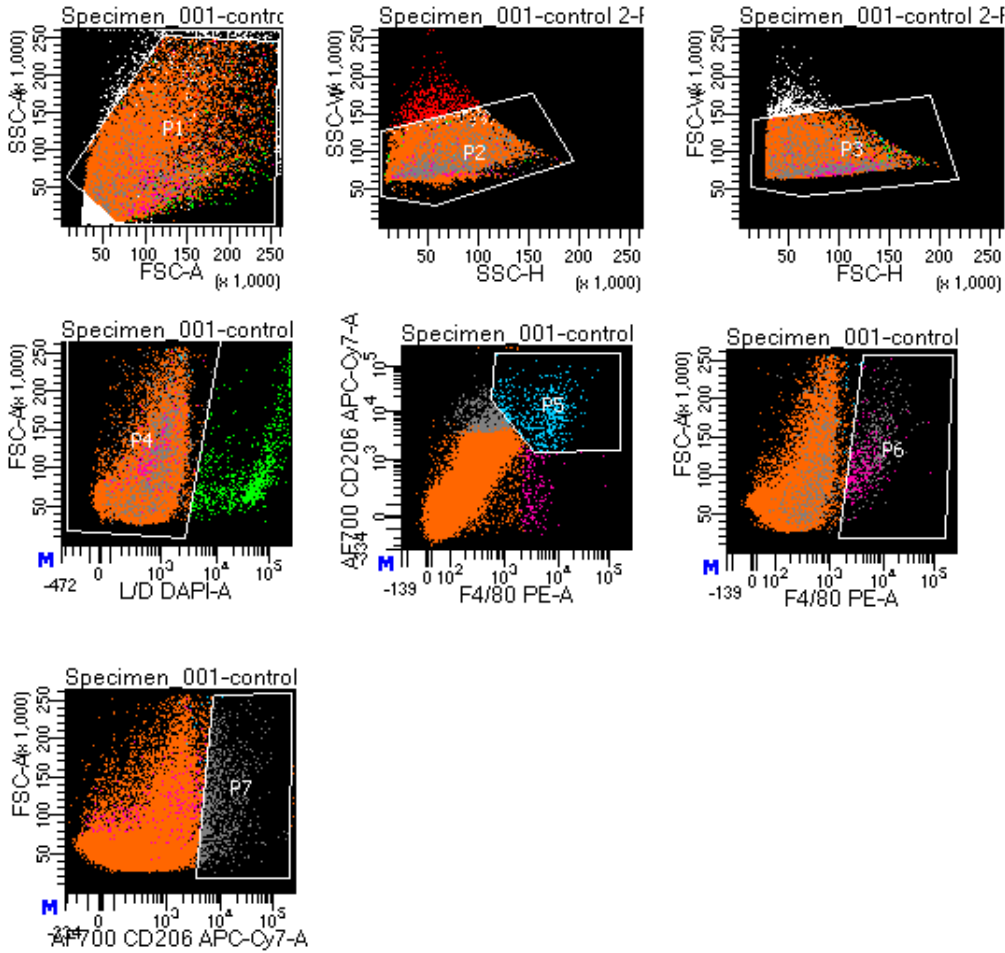

| Tube: control 2 |         |         |        |
|-----------------|---------|---------|--------|
| Population      | #Events | %Parent | %Total |
| All Events      | 50,000  | ####    | 100.0  |
| P1              | 33,992  | 68.0    | 68.0   |
| P2              | 33,451  | 98.4    | 66.9   |
| P3              | 33,050  | 98.8    | 66.1   |
| P4              | 32,292  | 97.7    | 64.6   |
| P5              | 473     | 1.5     | 0.9    |
| P6              | 523     | 1.6     | 1.0    |
| P7              | 801     | 2.5     | 1.6    |

Supplement: S21 Fig — FACS data for reparative macrophage isolation from heart 6 days port-MI. Gating strategies used for isolation of reparative (CD206+/F4/80+) macrophages from the 6 days post-MI hearts. (PDF) [file pbio.3000941.s021.pdf]
