# Supplementary material for: YAP/TAZ deficiency reprograms macrophage phenotype and improves infarct healing and cardiac function after myocardial infarction
Source: PLoS Biol. 2020 Dec 2;18(12):e3000941. doi: 10.1371/journal.pbio.3000941 (PMC7735680; doi:10.1371/journal.pbio.3000941)
Supplement: S11 Fig — (A) Reported-Tead binding sequences (TBSs). (B) Predicted TBSs in Arg1 promoter. (PDF) [file pbio.3000941.s011.pdf]

A

TGGAATG
AAGCATG
AAGTATG
TAGAATG
GGAATG
TGAATG

Reported Tead binding sites

B

Reported Tead binding sites in *Arg1* promoter
(3100bp promoter + exon1)

```
CTCTCAGAACCCATACAGCAAGGAGAACCTCCTCAGGGCCATGGTATGTGTTTCCCAACA
CGGACACACACACAATCACACAGAGTAAAGGGTTTTTTAATTAAGCAAGAAATACAGTTT
ACACATATGTCTGCATAAGGTCACGGAGGGTGGTAGCCGACGAGAGACCAGCTCATCTTC
AATAAGGAAGTCAGAGAGCAGAAGGCTTTGTCAGCAGGGCAAGACTATACTTTGTTAGGA
AGTGAGGCATTGTTCAGACTTCCTTATGCTTTCTTATGAACAGGCTGTATTAGCCAACAG
TCCTGTCTGTACGTGAGTTGTGCCACTTTGGGTTGAGGGTGCTGGGCTAACACAGATCTT
AGTTAAGCATTTCCTTGTCCTTTGATTCCTCAATGACCCAGAACAGGCAAACAATACGAT
ACTTATGTTTGATGAATTAGATGACTGCATCTACAACTGGACTTGTGCACTGAGTTCTGG
ATGGTCGCCTGAAGGAGAACGCAGGGAGGCAGGCGATACTTTAATAGGACTGACTGTGAG
GGTAAATCGGTGTCATACATGGAACCGTCGTGTGTATGGAGATACCCAGAACAATAAAGA
CCCAAGAAAAACAGATAGTCAAGAAATCATGAGTTTTACTTAATCAGGGCGTTTTGATCA
GTCAGTCCAATGATGCCAATGATGAAAAGTCACCCTCAAACTGCCCGAGGGAAGACTTTC
AGGAAAAGCCCTGGTGATAAAGTTTAACAGCAAATACCTATGAATGAATGTATAAAGCTA
GTAACGGCCGTAGCACTGTTTTCAAAACGTAGTCACCGGCTCACTCTACATTTGCGAGAC
CCCGACAGAAGTAAATGTAAGGTCAAGCGATTTTGAAAAAGAGGAGCACCAGTTCCTTTC
CTGAAGCTCTAACGTCCTCCGGAAGCCCTACCAATGAGGCTGCATGGATTGGTGCAGCCA
CAGGTGATGAGCCCAGGGCATAGAGGAAACTGGAAGATGGAGCAGCATATCGTTTATAAC
AGCAACTTGGCATCTGATGGAGATAGCTCAGTGGTAACGTGGGGAGTCTGTCCATTTGGT
GCTGGTGGGGAAACTGGGAGAGACAAAGGGGAAATGGAGGATTTAAACAAAATACAAAAA
AAAAAAAATGGACGCAACAGTCAGCAATCACTGTGAATAGCAAATTCTTCATGCATCCAA
GACTTAAGCCCAGCATCTTTCAGGCAGGAAAACAATCTACCCCTGAATGGCATTTTTCTT
CTTTTCTTTTTACCTTTGGGGCAGGTTCTGTGTTGACCTTTGAACTAACCCTATAACCCC
AGCAGGTGTTGAACCTATAATCCCCCTGCCTCATCCTCTCAAATAGCTGGAATTTGTGGG
CCAGCACCAAGAGGCCCAAAGAAAGAGAAAGTTTAAATTAAATAGAAGAAAATGTAGAGG
TATTGACAGGTGGTGGTATCACACACCTTTAATCCCAGCACTCAGGAGGCAGTGACAGAT
TGCTATCTGTGAGTTCAAGGTTAGCCTGGTCTATAGGGCTAGTTCTAGGGTAGCCAGAGC
CACACAAAGAAAACATGTCTCCAAAAAGCAAACAAAAATGTAGAGGTGCACATTTGGGAT
CTGGGAGCAGGTAAAACTTAAATTCCTAGCTAGCTTCAAATATATAGGAAAATTCTATCT
TACATAAATCAATTTTTTAAAAAAATCTATAACCGTATATCAGAAAAACATAGTAAGATG
AAAAATATATTTGAAAAGGAGGTATCAAAATGGTCCTGAGGGACTTTGGCTTCTTGATTT
ATTCATTCAATAAATGCAATACATTTATTCAATCAATAAGTCAATGTTTATTTTGTACTG
AACGATTCTATGTACTGATGGTAGAGGGAACAGAATCTTTCTTGAGGTATTAAAATTCTG
GGTGCTTTTTGTCCTCACAAGGTTAAAGAAATTTACTTCTGACATGTGGGTTTTCTTTTT
AAAATTATAATCATGTAACTTTGTAACTGGGAGAATACTTGGTTGTGTTTCTTCTAAATT
AATACTTTTAAAAATATTTCTTTAGTGTTGGGGGTGGCTTTACACAGGGACCGGACCACA
TCACCCACGTGGAGGTCAGAGATCAACTTGGAGGAGTTGACTCTTTCCTTGCACCAAATG
GGTTCTTCGGGTCAAACTCGGGTCATCGGGCTTGGTGTTACCGGCTGGCCTCTCTCATCT
GCCCTAGATCTCAGGGGGCCCTGCTGACACTCTGCTGGTGTGTAGATTAGAATCGCGGCA
GCTCGGCGGGTGCAGAATGCCCATTGCTCCGTTTCGATTCTTCTGCAACCTGTATGTGAC
TAGCAACCTCACCAAGCTGCAGTCTCTTCCTGGGCAAAACAGCATAAGAATGATTCTCAG
TCTGAAGTTAGTTTAAAGAGGAAACAGGAAGCGAAAGAACATTAACTAGAATAGCACTTG
GCACACGACAAAGACAACTCATATTAGCTAGTTTGCTTTCCCTATCTTGGGATATCATGA
TTCCAAAAATGAGATTTTCCTGGAGACTGTATTTCAAAAATAGAACTGCTTTGGGTTGTC
AGGGAAATAAATGATGCCTTCCTGTAAATAATAATAATAATAACAACAACAACAATATAA
AACAAAAACCAGCCCCATGCTTTCCTAGACAGTGTAACCTGGTGACACATGAAATGTGTC
TCACTTTCCCCAGAACTTGAAGCCTTGACTCAGGATGCTCAACAGGAGGGAAGTAAGAGA
CACCCCCCCCACCCCCTCACCCCCCCCCCCCACTTGCTGTTTTAGCCTCACCTTGCAAGT
CCCCAGTGGACTTAAATCCTGGAAAAGGTGAGCACCCTGCCCTGAGGTGCCCAGGCCGGA
AGCCTAGCACTTCACATGAGGTTATGAAATCACACATAATTGTCAATTGTCTGAGGAGAG
ATTAATGTCATCCAGCTGGCTTTTTCAAAAGGGTGTGAACTGGACGGATGAATAATGCTC
AGAGGGAAGAATGGTAGTTCCTCTGATGGGGAGGTTCTGTTGACTCTGTCATTCTTCCAT
TCGGTGGGCGGAGCCAGTTGTTGGATAAACAGATCCAACCTGATTATAAGGGGGGAAAAA
GATGTGCCCTCTGTCTTTTAGGGTTACGGCCGGTGGAGAGGAGCTGGACAGCCCGAGCAC
ATGCAGCAGCAGCAGCCGCTGGAACCCAGAGAGAGCATGAGCTCCAAGCCAAAGTCCTTA
GAGATTATCGGAGCGCCTTTCTCAAAAGGACAGGTGAGTTTGGCTTTGAATGACTGGAAT
```
