# Supplementary material for: YAP/TAZ deficiency reprograms macrophage phenotype and improves infarct healing and cardiac function after myocardial infarction
Source: PLoS Biol. 2020 Dec 2;18(12):e3000941. doi: 10.1371/journal.pbio.3000941 (PMC7735680; doi:10.1371/journal.pbio.3000941)
Supplement: S20 Fig — FACS data for proinflammatory macrophage isolation from heart 2 days port-MI. Gating strategies used for isolation of pro-inflammatory (iNOS+/F4/80+) macrophages from the 2 days post-MI hearts. (PDF) [file pbio.3000941.s020.pdf]

BD FACSDiva 9.0.1

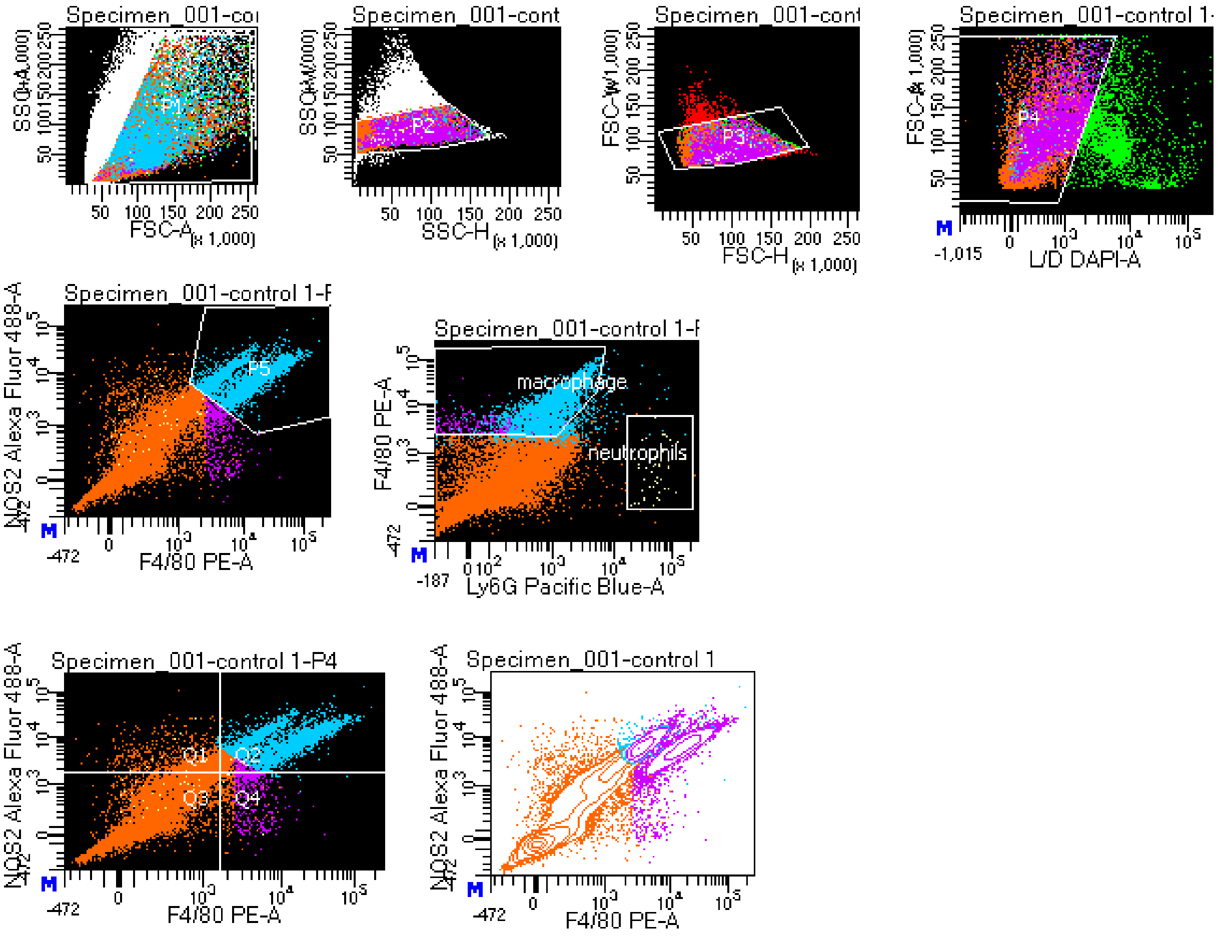

Tube: control 1

Population	#Events	%Parent	%Total
All Events	50,000	####	100.0
P1	26,765	53.5	53.5
P2	22,579	84.4	45.2
P3	21,920	97.1	43.8
P4	19,668	89.7	39.3
P5	3,237	16.5	6.5
macrophage	3,255	16.5	6.5
neutrophils	51	0.3	0.1
Q1	1,400	7.1	2.8
Q2	3,715	18.9	7.4
Q3	14,024	71.3	28.0
Q4	529	2.7	1.1
