# Supplementary material for: YAP/TAZ deficiency reprograms macrophage phenotype and improves infarct healing and cardiac function after myocardial infarction
Source: PLoS Biol. 2020 Dec 2;18(12):e3000941. doi: 10.1371/journal.pbio.3000941 (PMC7735680; doi:10.1371/journal.pbio.3000941)
Supplement: S9 Fig — (A–C) BMDMs were isolated from control and YAP/TAZ-dKO mice and stimulated with/without IL4/IL13 (M2a) or BSA/anti-BSA immune complex (M2b) or IL10/TGFβ1 (M2c) for 24 hours. Real-time qPCR was performed for (A) M2a (Ym1 and Cd206), (B) M2b (Il10 and Il1ra), and (C) M2c (Mmp9 and TGFβ) marker genes using RNA isolated from control and YAP/TAZ-dKO BMDMs. Data are shown as the mean ± SEM, n = 3 for each experimental group. Gene expression data were normalized with the reference gene Gapdh, and results are represented as fold change relative to the control treatment. For numerical raw data, please see S1 Data. (PDF) [file pbio.3000941.s009.pdf]

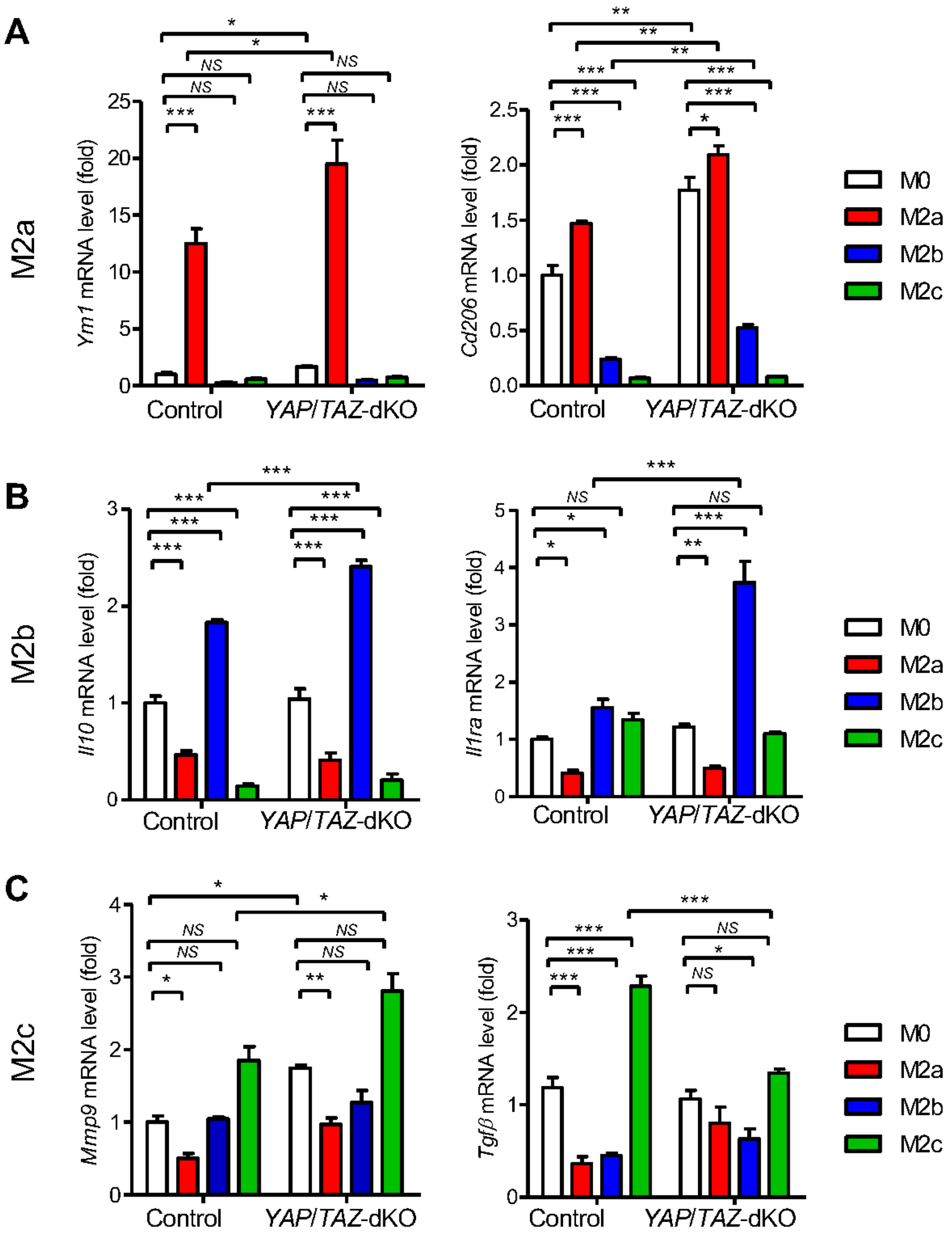

A
B
C
M2a
M2b
M2c
Ym1 mRNA level (fold)
Cd206 mRNA level (fold)
Il10 mRNA level (fold)
Il1ra mRNA level (fold)
Mmp9 mRNA level (fold)
Tgfβ mRNA level (fold)
Control
YAP/TAZ-dKO
NS
M0
M2a
M2b
M2c
