# Supplementary material for: YAP/TAZ deficiency reprograms macrophage phenotype and improves infarct healing and cardiac function after myocardial infarction
Source: PLoS Biol. 2020 Dec 2;18(12):e3000941. doi: 10.1371/journal.pbio.3000941 (PMC7735680; doi:10.1371/journal.pbio.3000941)
Supplement: Attachment — Submitted filename: Response to Reviewers.pdf [file pbio.3000941.s045.pdf]

Reviewer #1:

General comments:

Comment: In the submitted manuscript, Mia et al. investigate the functions of YAP/TAZ in myeloid cells following myocardial infarction. Using a bone marrow-derived macrophage model of macrophage differentiation, the authors provide evidence that YAP/TAZ are necessary and sufficient for optimal expression of genes associated with inflammatory responses (M1) and suppression of genes associated with a reparative (M2) macrophage phenotype. Mechanistically YAP/TAZ promoted inflammatory gene expression by directly binding to the IL-6 promoter and augmenting TLR4 signaling through a MAPK dependent pathway and impaired Arg1 expression (M2 marker) by interacting with the HDAC3-NCoR1 repressor complex. The authors explored the roles of YAP/TAZ in myeloid cells using an experimental mouse model of coronary ligation. Loss of YAP/TAZ in myeloid cells resulted in improved LV systolic function, reduced fibrosis, and increased coronary angiogenesis. Conversely, YAP activation led to exaggerated post-MI LV remodeling and increased myocardial fibrosis. The authors provide further evidence that manipulation of Hippo signaling in myeloid cells influenced macrophage gene expression. Overall, this manuscript is well written and the data are clearly presented. The finding that YAP/TAZ influence macrophage gene expression and potentially monocyte differentiation are novel and interesting.

Response: *We would like to thank the reviewer for his/her positive evaluation of our manuscript.*

Specific comments:

Comment: What is the biologically relevant signal that controls activation of YAP/TAZ in vivo? The authors provide data that macrophage activating stimuli (LPS/IFNg and IL4/13) result in increased YAP and TAZ mRNA and protein expression. Do the authors believe that modulation of YAP/TAZ expression is sufficient to control hippo signaling? As YAP/TAZ exert their effects within the nucleus, the authors should consider measuring nuclear YAP/TAZ protein abundance using fractionated cell preparations (nucleus vs. cytoplasm). The authors also comment on increased in phosphorylated YAP. It would be more ideal to measure non-phosphorylated YAP, which is the active form within the nucleus.

Response: *In the revised manuscript we show that hypoxia is one of the biological signals that can activate YAP/TAZ in macrophages. However, we cannot rule out other signals that may also activate YAP/TAZ in vivo. These results have been included in the revised manuscript (Supplementary Figure 16).*

YAP/TAZ are the mediator of the Hippo signaling pathway and not the regulator. The canonical Hippo pathway is a kinase cascade, wherein Mst1/2 kinases and Sav1 form a complex to phosphorylate and activate Lats1/2. Lats1/2 kinases in turn phosphorylate and inhibit the transcription co-activators YAP and TAZ, two major downstream effectors of the Hippo pathway. When dephosphorylated, YAP/TAZ translocate into the nucleus and interact with Tead1-4 and other transcription factors to induce gene expression. In the non-canonical Hippo pathway, activated Mst1/2 also phosphorylate substrates outside of the canonical Hippo pathway. The canonical/ non-canonical regulation of upstream and downstream components of the Hippo pathway might lead to alternative cellular outcomes during cardiac regeneration/repair. For instance, stress-induced activation of Mst1 leads to phosphorylation of Lats2, which form the canonical Hippo pathway together with YAP, and regulate cell proliferation (Heallen et al., 2013; Xin et al., 2013; Xin et al., 2011). Importantly, activated Mst1 also phosphorylate substrates outside of the canonical Hippo pathway, such as Bcl-xL proteins in the mitochondria, and promote cell death (Odashima et al., 2007; Yamamoto et al., 2003). Changes in the expression of YAP/TAZ suggest that the canonical pathway is affected during polarization. Previous work has demonstrated that deletion of upstream Hippo kinases Mst1/2

in myeloid cells leads to elevated expression of inflammatory genes including IL6 in a cecal-ligation-and-puncture (CLP) model of septic peritonitis (Geng et al., 2015). Here the focus of our study was to determine the role of Hippo signaling mediators YAP/TAZ in post-MI cardiac remodeling by modulating macrophage polarization.

We have prepared nuclear and cytoplasmic fractions using BMDMs and analyzed YAP, pYAP, and TAZ expression. These results are consistent with the previous observation that YAP/TAZ expressions are elevated during macrophage polarization. These results have been included in the revised manuscript (Supplementary Figure 1).

Comment: The authors conclude that YAP/TAZ potentiate inflammatory macrophage gene expression through two potential mechanisms: directly binding to the IL-6 promoter and augmentation of TLR4/TAK1/MAPK signaling. What is the relative contribution of each of these mechanisms? Does YAP/TAZ bind to the promoter of other inflammatory chemokines and cytokines that are regulated by YAP/TAZ? Does activated YAP potentiate TLR4 signaling in a TAK1/MAPK dependent manner? Functional data implicated the relative importance of these pathways are lacking. What is the mechanism by which YAP/TAZ influences TLR4/TAK1/MAPK signaling?

***Response:** We found that there was a significant reduction of phosphorylated Tak1 protein level in YAP/TAZ-dKO BMDMs after 15 minutes of LPS/IFNγ treatment (Figure 3B). Next, we tested the key components of MAPKs and observed significant but transient (only at 15 minutes time point) reduction in the levels of phosphorylated JNK, p38, and Erk1/2 in YAP/TAZ-deficient macrophages upon LPS/IFNγ stimulation. To determine the relative contribution of these two potential mechanisms regulating IL6 expression, we performed luciferase assay using IL6 reporter co-transfected with YAP plasmid in the presence or absence of Hippo signaling inhibitor verteporfin (VP) or MAPK inhibitor SB203580 (Supplementary Figure 8B-C). We observed a mild reduction in the luciferase activity in the presence of SB203580. In contrast, VP completely blocked the YAP medicated IL6 activation suggesting that direct regulation of IL6 by YAP may play a dominant role compared to the MAPK pathway in mediating IL6 activation (Supplementary Figure 8B-C).*

We see that changes in the TLR4/TAK1/MAPK signaling pathway are only transient. Quantification of western blot showed that changes are only significant at 15 minutes timepoint. Similarly, the luciferase data with MAPK inhibitor also showed minimal effect suggesting that the MAPK pathway has a limited role to play in YAP/TAZ mediated M1 macrophage polarization.

We have analyzed only some of the inflammatory gene's promoter in this study and identified multiple Tead binding sites within the 2kb IL6 promoter and that's why we have focused on it. However growing evidence suggests that many inflammatory genes are directly regulated by YAP/TAZ in immune cells as well in other cell types (Matsushita et al., 2019; Ramjee et al., 2017; Zhou et al., 2019). For example, TAZ directly binds to IL1β promoter and regulates its expression in Malignant mesothelioma cells (Matsushita et al., 2019). We have previously shown that YAP can directly bind and activate the IFNγ promoter in epicardial cells (Ramjee et al., 2017). We have also found that IL33 is a direct YAP/TAZ target in cardiac fibroblast (Unpublished observation).

Comment: The authors conclude that YAP/TAZ inhibits Arginase1 expression through an AKT dependent pathway that involves recruitment of the HDAC3-NCoR1 repressor complex to the arginase promoter. Functional data implicating AKT signaling is lacking. Does inhibition of AKT prevent assembly of this repressor complex? What is the mechanism by which YAP/TAZ deficiency augments AKT signaling? The HDAC3 siRNA experiments are not informative. A more ideal experiment would be to prevent the interaction between YAP and HDAC3.

Response: *We would like to clarify that we did not conclude that YAP/TAZ inhibits Arginase1 expression through an AKT dependent pathway that involves recruitment of the HDAC3-NCoR1 repressor complex to the arginase promoter. Activated PI3K/AKT pathway has also been recognized as an essential step towards M2-like polarization as its inhibition abrogates the upregulation of M2 polarization genes (Ruckerl et al., 2012). We, therefore, analyzed the levels of phosphorylated AKT in control and YAP/TAZ-dKO BMDMs treated with IL4/IL13. We observed a significant increase in phosphorylated AKT level in YAP/TAZ-deficient macrophages (Figure 4C). We show that YAP can physically interact with the HDAC3-NCoR1 complex and repress Arg1 promoter activity. We believe that changes in AKT levels just represent the M2 status of the macrophages after IL4/IL13 treatment. We have modified the text to reflect this in the revised manuscript. Overall, all the results presented in Figure 4 suggest that YAP/TAZ deficiency promotes M2 polarization.*

To further strengthen our data on YAP-HDAC3-NCoR mediated repression of Arg1 expression, we have performed multiple additional experiments and included the data in the revised manuscript. We demonstrate that the repressive response to Arg1 promoter-luciferase activity was further enhanced when HDAC3 was co-expressed together with YAP^{S127A} (a constitutively active form that remains in the nucleus and is transcriptionally active) compared to YAP (Figure 4K). We have also performed additional experiments to address the structural vs enzymic role of HDAC3 in the repression of Arg1 and included them in the revised manuscript. We observed that Vorinostat or Scriptaid treatment abolished the YAP-HDAC3 mediated repression of Arg1 (Figure 4O-P). Both Vorinostat or Scriptaid are known to affect both protein expression as well as the deacetylase activity of HDACs including HDAC3 (Banerjee et al., 2018; Meng et al., 2020; Sun et al., 2017). To further support our findings that the enzymatic function of HDAC3 is not essential for HDAC3-mediated repression of Arg1, we utilized an HDAC3-specific inhibitor RGFP966. In multiple biological systems, RGFP966 has been shown to affect the deacetylase activity of HDAC3 but not the protein expression (Leus et al., 2016; Suelves et al., 2017). We observed that RGFP966 treatment did not affect the YAP-HDAC3 mediated repression of Arg1 (Figure 4Q). To further demonstrate that the deacetylase activity of HDAC3 is not required for Arg1 repression, we utilized a previously described mutant form of HDAC3 in which two highly conserved tandem His residues, 134 and 135, are mutated to alanine ($HDAC3^{H134A, H135A}$). These mutations do not affect its expression, chromatin recruitment, and interaction with NCOR1; however, they render HDAC3 completely inactive (Lewandowski et al., 2015; Sun et al., 2013). We observed that $HDAC3^{H134A, H135A}$ mutant was able to repress the Arg1 reporter similar to HDAC3 suggesting that the deacetylase activity of HDAC3 is not required for Arg1 repression (Figure 4R).

Comment: The experiment myocardial infarction studies show clear and consistent phenotypes. However, the use of Lyz2-Cre is an important limitation as it is active in all myeloid cells including granulocytes. Thus, it is not possible to conclude that YAP/TAZ deficiency in monocytes and macrophages is responsible for the observed phenotypes. As many neutrophils are recruited to the heart following myocardial infarction, the authors should either use more selective Cre recombinases or provide evidence as to whether YAP/TAZ influence neutrophil phenotypes.

Response: *We have performed immunohistochemistry for Myeloperoxidase (MPO) on control and YAP/TAZ-dKO infarcted heart sections at 2 days post-MI. The number of MPO positive cells was determined in ≥5 distinct microscope fields for each heart section. At least four hearts were analyzed for each group. No differences in neutrophils recruitment were observed between control and YAP/TAZ-dKO group (Supplementary Figure 15A). Also, we also performed transwell migration assay using control and YAP/TAZ-dKO neutrophils. Cells that had migrated to the lower chamber of the transwell plate were visualized by MPO immunostaining. No difference in the number of migrated MPO^{+} cells was observed between the control and YAP/TAZ-dKO group (Supplementary Figure 15B). In addition to our findings, previous reports have demonstrated that the deletion of YAP using Lyz2-Cre does not affect neutrophils. The depletion of neutrophils did not affect the inflammatory phenotype observed*

(Zhou et al., 2019). In another study, authors demonstrated that YAP/TAZ double mutant bone marrow cells gave rise to differentiated cells of the myeloid and lymphoid lineages with the same efficiency of control bone marrow cells suggesting that YAP/TAZ are dispensable for adult hematopoiesis (Donato et al., 2018).

Comment: Presented echocardiographic data is limited to ejection fraction and fractional shortening. Given the asymmetric pattern of injury and left ventricular (LV) remodeling, fractional shortening is not an ideal measurement. The authors should include quantitative data pertaining to LV ejection fraction, LV diastolic and systolic volumes, and LV mass.

Response: *We have included additional quantitative data on LV ejection fraction, End Systolic Volume (LVESV), End Diastolic Volume (LVEDV), Stroke Volume (SV), LV mass and Fractional Area Change (FAC) (Figure 5 and Supplementary Figure 14).*

Comment: To assess whether changes in infarct size are a result of increased initial infarct area or infarct expansion, the authors should perform TTC staining within 24-48 hours of myocardial infarction to assess initial infarct size in each experimental group.

Response:
All the animal surgery was performed by an experienced technician (15 years) working at the National Heart Centre Singapore. He is also part of the animal core and performs surgery for all the laboratories. Nonetheless, to assess the initial infarct size we performed MI and first quantified the initial ischemic area in both control and YAP/TAZ-dKO hearts at days 2 post-MI. No significant change was observed between the two groups (Supplementary Figure 17A). Next, we also analyzed the fibrotic area by Masson's Trichome staining and observed no significant difference between the control and mutant groups at days 2 post-MI (Supplementary Figure 17B). We have included this data in the revised manuscript.

Comment: Pathological analysis of LV remodeling is limited to measurements of infarct size and coronary capillary density. The authors should also include quantification of cardiomyocyte cross sectional area within the border and remote zones as well as interstitial myocardial fibrosis.

Response: *We have performed Masson's trichrome staining on control and YAP/TAZ-dKO heart sections post-MI and analyzed as interstitial myocardial fibrosis. We observed a significant reduction in interstitial fibrosis in YAP/TAZ-dKO hearts compared to controls after MI (Supplementary Figure 18A-B). To determine the cardiomyocyte cross sectional area (CSA), we performed WGA staining and quantified the CSA within the border and remote zones. We observed a significant reduction in the CSA in YAP/TAZ-dKO hearts compared to the controls (Supplementary Figure 18C-D). We also performed qPCR analysis for some of the cardiac hypertrophy marker genes and found that the levels of Nppa, Nppb, and Myh7 were reduced in YAP/TAZ-dKO hearts compared to the controls (Supplementary Figure 18E).*

Comment: A key conceptual limitation of this manuscript is the focus on M1 vs. M2 macrophages. This is an outdated classification of macrophages that is derived from in vitro polarization studies. A more modern view is that monocytes have the capacity to differentiate into a variety of macrophage subtypes with phenotypes that range between the extremes formulated in the M1 and M2 designations. Markers of in vitro derived M1 and M2 macrophages rarely coincide with in vivo populations. Furthermore, the heart contains both resident macrophages and recruited monocyte-derived macrophages that are not described by the M1/M2 classification. While the authors mRNA expression data evaluating macrophage gene expression are informative, the quantification of NOS2+ and CD206+ macrophages is less helpful. Differences between CD206+ macrophages seem to be driven by 2 outlying data points. If the authors wish to make rigorous conclusion regarding monocyte and macrophage differentiation, it would be more helpful to partition macrophages into established subsets such as CCR2-/LYVE1+ and CCR2+/LYVE1- populations or use single cell RNA sequencing.

Response: *We agree with the reviewer that monocytes can differentiate into a variety of macrophage subtypes and macrophages show a dynamic transition between two polarization states, M1 (pro-inflammatory) and M2 (anti-inflammatory). In some pathological conditions, macrophages can develop mixed M1/M2 phenotypes (Pettersen et al., 2011; Vogel et al., 2013). However these states are not well characterized and their independent functions in regulating inflammation/repair are not established (Dick et al., 2019; Mouton et al., 2018; Rizzo G, 2020). To determine the further M2 subtypes, we isolated BMDMs from control and YAP/TAZ-dKO mice and stimulated with/without IL4/IL13 (for M2a) or BSA/anti-BSA immune complex (for M2b) or IL10/TGFβ1 (for M2c) for 24 hours. Real-time qPCR was performed for M2a (Ym1 and Cd206), M2b (Il10 and Il1ra), and M2c (Mmp9 and TGFβ) marker genes using RNA isolated from control and YAP/TAZ-dKO BMDMs (Supplementary Figure 9). We observed elevated levels of these markers suggesting that YAP/TAZ deletion leads to M2 polarization.*

Recent publications from multiple independent cardiac group do not support the comment that "markers of in vitro derived M1 and M2 macrophages rarely coincide with in vivo populations". For example, it has been shown that M2-like macrophages (CD206+F4/80+) are present in the left ventricular myocardium of adult mice heart at 7 days post-MI. These CD206+F4/80+ cells expressed high levels of M2 macrophage markers such as CD206, Ym1, and Fizz1 (Shiraishi et al., 2016). Likewise, a recent study has defined the macrophage population as CD206 negative (M1) or CD206 positive (M2) in post-MI hearts (Barnette et al., 2018). Similarly, NOS2+/F4/80+ macrophages were considered as M1 macrophages to determine the M1 population in total macrophages (Chen et al., 2018; Peng et al., 2019; Sun et al., 2019; Tan et al., 2019; Xu et al., 2016). Gene expression analysis on Isolated macrophages from other non-cardiac pathological tissues also demonstrates similarity in in vivo and in vitro M1/M2 marker genes. We agree with the reviewer however that not all the in vitro markers correlate with the in vivo markers (Barnette et al., 2018; Han et al., 2020; Mouton et al., 2018; Rizzo G, 2020).

We agree with the reviewer that the heart contains both resident macrophages and recruited monocyte-derived macrophages in steady-state. However, the majority of the resident macrophages are depleted after MI and rapidly replaced by monocytes in the injured heart. Post-MI cardiac inflammation and remodeling is primarily driven by infiltrating monocytes (Dutta and Nahrendorf, 2015; Epelman et al., 2014; Heidt et al., 2014; Nahrendorf et al., 2007; Pinto et al., 2014; Pinto et al., 2012). We are not trying to make rigorous conclusion regarding monocyte and macrophage differentiation. Our goal was just to show the relative presence of inflammatory and reparative macrophage populations in the control and YAP/TAZ-dKO hearts port-MI. We believe this variation in CD206+ macrophage populations is due to sample preparation as tissue fibrosis in post-MI can affect the preparation of the single-cell suspension. However, the difference between the control and YAP/TAZ-dKO group is highly significant. We have revised the text to reflect this.

Our goal was to show that pro-inflammatory genes are down-regulated and anti-inflammatory/reparative genes are upregulated in YAP/TAZ-dKO. Based on our data and recent literature published in multiple competitive journals including PLOS family journals (PLoS Biology, PLoS Pathogen, PLoS Computational Biology, Cell Metabolism, Immunity, JCI, Science Advances, PNAS, Nature Communications, Cell Reports, JCI Insight, etc.) using M1/M2 classification, we thought it will be easier to convey the message following the M1/M2 classification (Arora et al., 2019; Carestia et al., 2019; Celik et al., 2020; Horhold et al., 2020; Liu et al., 2019; Liu et al., 2020; Petty et al., 2019; Sarode et al., 2020; Tuladhar et al., 2019; Ueta et al., 2019; Yu et al., 2019; Zhang et al., 2020a; Zhang et al., 2020b; Zhao et al., 2019; Zhou et al., 2019). Considering the reviewer's comment, we are happy to use the terms pro-inflammatory and anti-inflammatory/reparative phenotypes instead of M1/M2.

Reviewer #2:

General comments:

Comment: In their paper, Mia et al. show how differentiation of bone marrow-derived macrophages (BMDMs) to pro-inflammatory (M1) or anti-inflammatory (M2) macrophages is regulated in vitro and after myocardial infarction (MI). After MI, ineffective recovery and adverse cardiac remodeling initiate structural and also functional changes in the heart and are the cause of developing heart failure. Thereby, a complex inflammation cascade regulates the initial pro-inflammatory M1-mediated response as well as the following anti-inflammatory response, which is mediated by M2 macrophages and supposed to be the reparative phase. The rational of this study was to find new regulators of macrophage polarization and post-MI inflammation. The authors concentrated on YAP and TAZ, two central transcriptional co-factors of the Hippo signalling pathway. They can show that YAP/TAZ expression is increased in BMDMs during M1 or M2 polarization and that in YAP/TAZ-dKO mice (Myeloid specific YAP/TAZ knock-out), M1 polarization is impaired while M2 polarization is favoured leading to reduced fibrosis, increased angiogenesis and improved heart function 28d post-MI. This is mediated by interaction of YAP with the IL-6 promotor and the HDAC3-NCoR1 repressor complex. These results may render YAP/TAZ as future therapeutically targets in enhancing the anti-inflammatory response after MI. The study is well conducted and interesting, although the shown interaction of YAP/TAZ with the Hippo signalling pathway is not novel (as already mentioned in the references; Zhou et al., 2019). The novelty is the finding that the same mechanism is responsible for macrophage differentiation after MI and the YAP/TAZ interaction with HDAC3-NCoR1 complex.

Response: *We would like to thank the reviewer for his/her positive evaluation of our manuscript.*

Specific comments:

Comment: Fig. 1A and B: The proposed activation of YAP after LPS/IFNγ stimulation by enhanced phosphorylation of S127 is hard to see in the depicted western blots and blots either should be changed or removed (Fig. 1A). In addition, the increase in IL-6 expression in BMDMs is not convincing. The exposure of the blot showing Actin expression in PMs is too long and should be reduced. Expression of IL-10, VEGF or TGF-ß should be shown to exclude differentiation to M2 macrophages. Please also include quantification of all Western Blots.
Comment: Fig. 1E: It would be interesting to see protein expression data of IL-6 and IL-10, VEGF or TGF-ß. This could further help to evaluate the differentiation pattern.

Response: *We have changed the pYAP, IL6, and β-actin blots in the revised manuscript (Figure 1A-B). Also, we have prepared nuclear and cytoplasmic fractions using BMDMs and analyzed YAP, pYAP, and TAZ expression (Supplementary Figure 1). These results are consistent with the observation that YAP/TAZ are activated during macrophage polarization.*

We have further characterized the BMDMs and PMs to determine their differentiation pattern. BMDMs and PMs were isolated from wild-type mice and stimulated with/without LPS/IFNγ (M1 stimuli) for 0, 4, 8, and 12 hours respectively. Western blot analysis was performed for TGFβ (M2 marker) using cell lysates of BMDMs and PMs. The relative expression was quantified and no significant difference was observed (Supplementary Figure 2A). However, we observed a reduction in the RNA levels of Tgfβ and Vegf after 12 hours of treatment (Supplementary Figure 2B). These results suggest that LPS/IFNγ (M1 stimuli) does not induce M2 differentiation. Similarly, BMDMs were isolated from wild-type mice and stimulated with/without IL4/IL13 (M2 stimuli) for 0, 4, 8, and 12 hours respectively. Western blot analysis for IL6 (M1 marker) was performed using cell lysates of BMDMs. The relative expression was quantified and no significant difference was observed suggesting that IL4/IL13 (M2 stimuli) does not induce M1 differentiation (Supplementary Figure 2C). We did not analyze the expression of

IL10 as it is not a good M2 marker as many studies have shown that its expression is induced even with LPS/IFNγ (M1 stimuli) treatment (Al-Shaghdali et al., 2019; Baseler et al., 2016; Choi et al., 2016; Iyer et al., 2010; Matsumura et al., 2012).

All the blots have been quantified in the revised manuscript.

Comment: Fig. 1C: Results should be shown at least in duplicates and be quantified. There is still a significant expression of IL-6 in YAP depleted BMDMs. Does that mean a subpopulation of BMDMs nevertheless differentiated to M1 macrophages? This is also confirmed by the reduction of M1 marker gene expression by only around 20% compared to control siRNA with stimulation (Fig. 1D). Western blot data for Control siRNA without stimulation should be included. In general, do the authors have data regarding siRNA mediated TAZ downregulation in BMDMs or data showing effects of YAP/TAZ downregulation in PMs? Or are the observed effects restricted to BMDMs and circulating macrophages, respectively?

Response: *The siRNA experiment presented in Figure 1C was repeated at least 3 times. We have quantified all the blots and included the data in the revised figure. YAP is not the only regulator of IL6 in macrophages. Our both qPCR and western data suggest that there is a significant reduction in IL6 expression in YAP-KD or YAP/TAZ-dKO BMDMs. The expression of IL6 or other cytokines is not abolished due to YAP/TAZ deletion. Our findings are consistent with recent observations in Bowel and liver diseases (Song et al., 2020; Zhou et al., 2019). In unstimulated conditions, YAP expression is very low and it will be impossible to see a difference in expression after knockdown that's why we stimulated these cells (Figure 1A, B, D-E). We have provided new data from TAZ knockdown in BMDMs. Consistent with other findings we do observe reduced expression of M1 (IL6, IL1b, and Nos2) and increased expression of M2 marker genes (Arg1, Ym1, and Fizz1) (Supplementary Figure 3). Our findings suggest a role for YAP/TAZ in BMDMs and circulating macrophages. However, work from others has shown that the effects of YAP/TAZ deletion are observed in all the macrophages. For example, a recent study by Zhou et al demonstrated the role of YAP in PM causing Bowel disease (Zhou et al., 2019). Another study demonstrated a similar role for YAP in liver Kupffer cells (Song et al., 2020).*

Comment: Supp. Fig. 4 and 5: Results showing data of macrophages in an activated state would strengthen the hypothesis that Yap/TAZ-dKO did not affect proliferation or migration properties of BMDMs.

Response: *In supplementary Figures 4 and 5, experiments were performed on activated cells (LPS/INFγ treated). We have clarified this in the revised manuscript. Our results that the deletion of YAP/TAZ does not affect the proliferation or migration of the activated BMDMs (Supplementary Figure 6). Our results are consistent with two previously published papers (Donato et al., 2018; Zhou et al., 2019).*

Comment: Fig. 3 and 4: Of much interest would be the effect of YAP/TAZ-dKO in BMDMs on proliferation and migration. This could include a rescue experiment e.g. with recombinant IL-6.

Response: *We have performed proliferation and migration assay and found that the deletion of YAP/TAZ does not affect the proliferation or migration of the BMDMs (Supplementary Figure 6). Our results are consistent with two previously published papers (Donato et al., 2018; Zhou et al., 2019). We do not think that inflammatory changes seen due to YAP/TAZ deletion will affect its ability to proliferate or migrate.*

Comment: Fig. 3B and C: The reduced protein expression of P-Tak1 and of the key components of MAPKs is only seen after 15min of LPS/INFγ treatment. 30min after stimulation expression pattern are equal compared to control. Furthermore, since phosphorylation is a posttranslational modification, it cannot be measured with qRT-PCR. Here, total mRNA

expression is analysed and again only 15min after stimulation there is a difference in mRNA expression. That could explain the increase in phosphorylated protein in that time point. That stresses the point that YAP/TAZ-dKO in BMDMs affects a mechanisms or signalling pathway upstream of MAPKs and Tak1 axis. Since total mRNA expression is also affected as seen in the qRT-PCR results western blot data should be included in Fig. 3B.

Response: *We have quantified the blots and we see the most significant changes at 15 min time point. We apologize for not making it clear. Figure 3C is the quantification of blots used in Figure 3B. In the revised manuscript, we have re-organized the figure and modified the text to make it clear.*

Comment: Fig. 3: Do the authors have data regarding a direct promotor binding of other M1 marker genes depicted in Fig. 1D? Does YAP only binds IL-6 promotor directly? How about TAZ?

Response: *We have not analyzed all the M1 gene (inflammatory genes) promoter in our study. However growing evidence suggests that many inflammatory genes are directly regulated by YAP/TAZ in immune cells as well in other cell types (Matsushita et al., 2019; Ramjee et al., 2017; Zhou et al., 2019). For example, TAZ directly binds to IL1β promoter and regulates its expression in Malignant mesothelioma cells (Matsushita et al., 2019). We have previously shown that Yap can directly bind and activate the IFNγ promoter in epicardial cells (Ramjee et al., 2017). We have also found that IL33 is a direct YAP/TAZ target in fibroblast (Unpublished observation). We analyzed the promoter and identified multiple Tead binding sites within the 2kb IL6 promoter and that's why we have followed it up. We have performed ChIP assay with TAZ and found that it can also bind to the IL6 promoter in BMDMs (Figure 3F).*

Comment: Fig. 4B and C: Actin blots are exposed too long. Western blots should be quantified.

Response: *We have replaced the actin blots. All the blots have been quantified in the revised manuscript.*

Comment: Fig. 4I: Please also include data for WB: Myc and the respective band sizes in all blots. Are data available showing IP: Flag with WB: Myc?

Response: *We have included data for WB: Myc (Figure 4L). We have also included data showing IP: Flag with WB: Myc (Figure 4L). Band sizes have been included in all the blots. All these results have been included in the revised manuscript.*

Comment: Fig. 5 and 6: It is not clear whether the infarct region or remote region is the focus of investigation here. Please indicate.

Response: *We have changed the figure legends based on the focus of the investigation and we have included this information in the revised manuscript.*

Comment: Fig. 5E: There is a huge variance in the macrophage subtype population 6d post-MI in the YAP/TAZ-dKO group. Do the authors have any explanation for that? Please also include flow cytometry analysis of M1 and M2 macrophages of all in Fig. 5F-I indicated time points.

Response: *We believe this variation is due to sample preparation as tissue fibrosis in post-MI can affect the preparation of the single-cell suspension. However, the difference between the control and YAP/TAZ-dKO group is highly significant. We would like to clarify that results presented in F and G are from the sorted cells. Gene expression changes presented in figure 5H and I are from the heart tissues. We have included the flow cytometry analysis of M1 and M2 macrophages in the revised manuscript (Supplementary Figure 5).*

Comment: Fig. 6A and 7G: How was the measured fibrotic area normalized? Normalized to the remaining heart or to the left ventricle? Furthermore, all heart section should be analysed at the level of the papillary muscle. This is not evident by the depicted pictures.

Response: *The images presented here are just representative. The amount of post-MI myocardial fibrosis in control and mutant mice was quantified using ImageJ. Briefly, serial sections starting from the coronary ligature to the cardiac apex were stained with Masson's trichrome to determine the fibrotic tissue. At least five cross-sectional images for each heart were analyzed for blue myocardium (fibrotic tissue), followed by the total heart. Statistical differences in the percentage of fibrosis were determined with the unpaired t-test. We have included this information in the method section of the revised manuscript.*

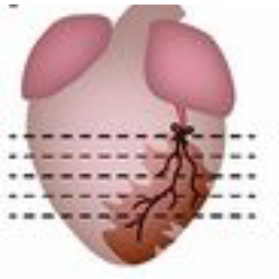

Comment: Fig. 7G: Please also include echo data.

Response: *We have not able to perform any animal surgery/echo work since the Covid-19 epidemic started. Our surgeon went to China for the Chinese new year and got stuck there since late January 2020. We have not been able to recruit a replacement due to the travel restrictions imposed currently. Our initial goal was to see whether the fibrotic phenotype is exacerbated due to Yap activation. The results presented in Figure 7 are supportive of the rest of the data presented in the manuscript and do not draw an independent conclusion. We appreciate your understanding of this unexpected situation.*

Comment: Please also conduct and include an early echocardiography time point in Figure 5 and Figure 7 to exclude (or show) an initial difference in infarct size between Yap/Taz knock-out, control and YAP overexpressing mice. Alternatively, Serum Troponin levels could be determined to demonstrate similar initial myocardial injury.

Response: *All the animal surgery was performed by an experienced technician (15 years) working at the National Heart Centre Singapore. He is also part of the animal core and performs surgery for all the laboratories. To determine the initial myocardial surgery, we performed MI and quantified the ischemic area. We also analyzed the fibrotic area by Masson's Trichome staining. No significant difference was observed between the control and mutant groups. We have included this data in the revised manuscript (Supplementary Figure 17).*

Reviewer #3:

General comments:

Comment: Using a range of elegant techniques that span, the authors show differential regulation of M1 and M2 macrophages by YAP/TAZ. Specifically, YAP/TAZ is required for M1 polarisation whereas it is inhibitory to M2 activation. The authors identify the targets of YAP/TAZ in these different macrophage states and identify that Ncor/HDAC repressor complex participates in the inhibition of gene expression by YAP. Notably, through decreasing M1 macrophages, YAP/TAZ deletion enhances post-MI remodelling and reduces fibrosis. This story is generally sound and the data is internally consistent. The findings provided add to the increasing knowledge of the YAP/TAZ pathway in the heart as well as other organs. These findings are of significance and contribute to our knowledge of how M1 and M2 macrophages are regulated in the heart.

Response: *We would like to thank the reviewer for his/her positive evaluation of our manuscript.*

Specific comments:

Comment: The authors rule out the NFkB pathway as a mechanism for activation of IL6 expression based on lack of phosphorylation of IKKαβ. The sensitivity of this assay may not be sufficient to rule out its role. I would suggest analysis of a luciferase reporter of NfkB signalling.

Response: *We have performed luciferase assay by using a commercially available NF-kB-Luc reporter (Promega, Cat No. E8491) and observed no changes when co-transfected with YAP expression plasmids suggesting that NF-kB signaling has no or very limited role if any in YAP mediated activation of IL6 (Supplementary Figure 8A).*

Comment: The effects of YAP/TAZ DKO on the expression of M1 markers revealed by qRT-PCR are not particularly dramatic. While these effects of YAP-TAZ KO are significant, in terms of absolute effects on induction they are quite small, cf. Rantes from a 300 to 200 fold induction? Yet, an end biological effect is reported – albeit small. Can the abundance of these cytokines be directly measured. How does this change in mRNA relate to protein? I have similar concerns re Fig 4A and Fig 7A. RE Fig 4A, the absolute changes in expression of M2 marker induction by YAP/TAZ KO are relatively small. Is the effect substantial enough to explain the biology proposed? In Fig 7 A, we see the effects of overexpression of a constitutively active YAP. Yet again, the relative changes in message are small.

Response: *We agree with the reviewer that changes are not dramatic but they are all significant. In our RNAseq screen, we observed hundreds of genes changing due to loss of YAP/TAZ and have focused on a subset of M1 and M2 marker genes in this study for in-depth analysis (Figure 2A). We believe that the accumulative changes observed in YAP/TAZ-dKO are sufficient to cause the biological effects on cardiac function, remodeling and repair observed. These changes are also consistent with recent observations by other groups in other biological systems (Geng et al., 2015; Song et al., 2020; Zhou et al., 2019). We apologies if it was not a clear presentation on our part but we do not think that the biological effects observed are small. In YAP/TAZ-dKO mice, we observed 32.41% improvement in left ventricular ejection fraction (LVEF), 27.04% improvement in fractional shortening (FS), 43.5% reduction in left ventricular end-systolic volume (LVESV), 25.78% improvement in fractional area change (FAC) and 40.47% increase in stroke volume (SV), compared to the controls post-MI (Figure 5 and Supplementary Figure 14). These changes are considered a very significant improvement of cardiac function in the post-MI heart. We have included more functional data and modified the text to reflect this in the revised manuscript. We have shown both at mRNA*

and protein levels that expression of M1 (IL6, IL1β, and Nos2) and M2 (Arg1 and TGFβ) marker genes are altered due to YAP/TAZ deletion.

Comment: These qRT-PCR data were normalised against one reference gene, GAPDH. Normalising to one reference gene is not best practice. Was this reference gene deemed to be stable between all conditions analysed. If so, provide the data to show its expression relative to a panel of other reference genes.

Response: *At the beginning of the study we determined the expression of both GAPDH and β-actin between all conditions analysed. We observed that GAPDH expression was stable between all conditions analysed so we decided to use it. At the protein level, we did not observed any difference in the expression of GAPDH or β-actin between all conditions analysed.*

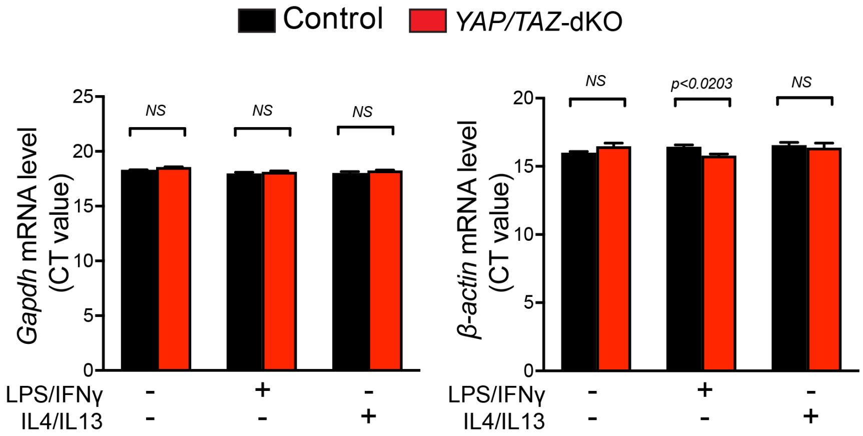

Comment: The authors indicate that that YAP/TAZ interacts with the HDAC3-NCoR1 complex to repress Arg1 expression in macrophages. In support of this, they show that by IP experiments the interaction of the proteins in this complex and that YAP directly binds to the promoter of the ARg1. It was not clear to me whether experiments were conducted to test a direct interaction of YAP with DNA. The authors go on to show that the transfection of HDAC represses the expression of this reporter. The observation that this Luc reporter is transiently transfected and not chromatinised would make it surprising that the repressor complex was operating in such a manner. I do not rule out this possibility however, especially if the repression is not via alterations in histone acetylation but via a direct protein mediated repression. Experiments to identify the interaction domain between YAP and the repressor complex which could then be modified to prevent such an interaction would substantially increase the weight of evidence in favour of a functional interaction between YAP and the repressor complex.

Response: *We did not test a direct interaction of YAP with DNA because YAP/TAZ are transcriptional coactivators and lack a DNA-binding domain but contains an activation domain. They interact with TEAD1-4 proteins (which contain a DNA-binding domain but lack an activation domain) and form functional heterodimeric transcription factors to activate target gene expression (Huang et al., 2005; Kim et al., 2018; Tian et al., 2010). To further strengthen our data on YAP-HDAC3-NCoR mediated repression of Arg1 expression, we have performed multiple additional experiments and included the data in the revised manuscript. We demonstrate that the repressive response to Arg1 promoter-luciferase activity was further enhanced when HDAC3 was co-expressed together with YAP^{S127A} (a constitutively active form that remains in the nucleus and is transcriptionally active) compared to YAP (Figure 4K). We have also performed additional experiments to address the structural vs enzymic role of HDAC3 in the repression of Arg1 and included them in the revised manuscript. We observed that Vorinostat or Scriptaid treatment abolished the YAP-HDAC3 mediated repression of Arg1 (Figure 4O-P). Both Vorinostat or Scriptaid are known to affect both protein expression as well*

as the deacetylase activity of HDACs including HDAC3 (Banerjee et al., 2018; Meng et al., 2020; Sun et al., 2017). To further support our findings that the enzymatic function of HDAC3 is not essential for HDAC3-mediated repression of Arg1, we utilized an HDAC3-specific inhibitor RGFP966. In multiple biological systems, RGFP966 has been shown to affect the deacetylase activity of HDAC3 but not the protein expression (Leus et al., 2016; Suelves et al., 2017). We observed that RGFP966 treatment did not affect the YAP-HDAC3 mediated repression of Arg1 (Figure 4Q). To further demonstrate that the deacetylase activity of HDAC3 is not required for Arg1 repression, we utilized a previously described mutant form of HDAC3 in which two highly conserved tandem His residues, 134 and 135, are mutated to alanine (HDAC3$^{H134A, H135A}$). These mutations do not affect its expression, chromatin recruitment, and interaction with NCOR1; however, they render HDAC3 completely inactive (Lewandowski et al., 2015; Sun et al., 2013). We observed that HDAC3$^{H134A, H135A}$ mutant was able to repress the Arg1 reporter similar to HDAC3 suggesting that the deacetylase activity of HDAC3 is not required for Arg1 repression (Figure 4R).

Comment: Subsequent experiments in which HDAC3 has been reduced in expression by siRNA may also provide misleading results. HDAC would of course repress the expression of many genes, some of which may not be related to the endpoints studied in this MS. Some of these effects will be via protein/protein interactions which will be disrupted by KD. I do not understand why supporting experiments using an inhibitor of HDAC were also not performed. These would rule in/out the role of HDAC activity and could contribute to the understanding of the structural vs enzymic role of HDAC in repression of Arg.

Response: *Thank you for these suggestions. We have performed additional experiments to address the structural vs enzymic role of HDAC3 in the repression of Arg1 and included them in the revised manuscript. We observed that Vorinostat or Scriptaid treatment abolished the YAP-HDAC3 mediated repression of Arg1 (Figure 4O-P). Both Vorinostat or Scriptaid are known to affect both protein expression as well as the deacetylase activity of HDACs including HDAC3 (Banerjee et al., 2018; Meng et al., 2020; Sun et al., 2017). To further support our findings that the enzymatic function of HDAC3 is not essential for HDAC3-mediated repression of Arg1, we utilized an HDAC3-specific inhibitor RGFP966. In multiple biological systems, RGFP966 has been shown to affect the deacetylase activity of HDAC3 but not the protein expression (Leus et al., 2016; Suelves et al., 2017). We observed that RGFP966 treatment did not affect the YAP-HDAC3 mediated repression of Arg1 (Figure 4Q). To further demonstrate that the deacetylase activity of HDAC3 is not required for Arg1 repression, we utilized a previously described mutant form of HDAC3 in which two highly conserved tandem His residues, 134 and 135, are mutated to alanine (HDAC3$^{H134A, H135A}$). These mutations do not affect its expression, chromatin recruitment, and interaction with NCOR1; however, they render HDAC3 completely inactive (Lewandowski et al., 2015; Sun et al., 2013). We observed that HDAC3$^{H134A, H135A}$ mutant was able to repress the Arg1 reporter similar to HDAC3 suggesting that the deacetylase activity of HDAC3 is not required for Arg1 repression (Figure 4R).*

Comment: The authors provide data on EF and FS for heart function. When performing 2D echo, EF and FS are derived from the same measures and are thus not independent. The one reflects the other. Using the one measure to support the other is not appropriate. Based on both measures the effect of YAP KO are not substantial. It would be useful to have other measures of cardiac remodelling. For example, are there any differences in myocytes size, wall thickness, expression of fetal genes? Further and to support the contention that fibrosis is affected by YAP KO, a measure of strain or relaxation velocity would be useful. This would tell us the stiffness of the ventricle.

Response: *We have included additional quantitative data pertaining to LV ejection fraction, fractional shortening (FS), end-systolic volume (LVESV), End diastolic volume (LVEDV), Stroke volume (SV), LV mass and Fractional area change (FAC) (Figure 5 and Supplementary Figure 14). Increased FAC suggests that YAP/TAZ-dKO hearts are working more efficiently than the controls post-MI. To determine cardiac remodelling, we have performed Masson's*

trichrome staining on control and YAP/TAZ-dKO heart sections post-MI and analyzed as interstitial myocardial fibrosis. We observed a significant reduction in interstitial fibrosis in YAP/TAZ-dKO hearts compared to controls after MI (Supplementary Figure 18A-B). To determine the cardiomyocyte cross sectional area (CSA), we performed WGA staining and quantified the CSA within the border and remote zones. We observed a significant reduction in the CSA in YAP/TAZ-dKO hearts compared to the controls (Supplementary Figure 18C-D). We also performed qPCR analysis for some of the cardiac hypertrophy marker genes and found that the levels of Nppa, Nppb, and Myh7 were reduced in YAP/TAZ-dKO hearts compared to the controls (Supplementary Figure 18E).

Comment: IN Fig 6G the authors analyse the expression of endmucin in heart sections as a measure of capillary density. The data provided is a surface area coverage of this marker. It is not capillary density, which is the more often used measure. The authors should analyse for e,g. CD31 staining in tissue sections.

Response: *We have analyzed the vascularization of the infarcted control and YAP/TAZ-dKO hearts with CD31 as well as Lectin immunostaining. The results are consistent with previous observations suggesting improved vascular growth (Supplementary Figure 19).*

References:

Al-Shaghdali, K., Durante, B., Hayward, C., Beal, J., and Foey, A. (2019). Macrophage subsets exhibit distinct E. coli-LPS tolerisable cytokines associated with the negative regulators, IRAK-M and Tollip. PLoS One *14*, e0214681.

Arora, H., Wilcox, S.M., Johnson, L.A., Munro, L., Eyford, B.A., Pfeifer, C.G., Welch, I., and Jefferies, W.A. (2019). The ATP-Binding Cassette Gene ABCF1 Functions as an E2 Ubiquitin-Conjugating Enzyme Controlling Macrophage Polarization to Dampen Lethal Septic Shock. Immunity *50*, 418-431 e416.

Banerjee, N.S., Moore, D.W., Broker, T.R., and Chow, L.T. (2018). Vorinostat, a pan-HDAC inhibitor, abrogates productive HPV-18 DNA amplification. Proc Natl Acad Sci U S A *115*, E11138-E11147.

Barnette, D.N., Cahill, T.J., Gunadasa-Rohling, M., Carr, C.A., Freeman, M., and Riley, P.R. (2018). iRhom2-mediated proinflammatory signalling regulates heart repair following myocardial infarction. JCI Insight *3*.

Baseler, W.A., Davies, L.C., Quigley, L., Ridnour, L.A., Weiss, J.M., Hussain, S.P., Wink, D.A., and McVicar, D.W. (2016). Autocrine IL-10 functions as a rheostat for M1 macrophage glycolytic commitment by tuning nitric oxide production. Redox Biol *10*, 12-23.

Carestia, A., Mena, H.A., Olexen, C.M., Ortiz Wilczynski, J.M., Negrotto, S., Errasti, A.E., Gomez, R.M., Jenne, C.N., Carrera Silva, E.A., and Schattner, M. (2019). Platelets Promote Macrophage Polarization toward Pro-inflammatory Phenotype and Increase Survival of Septic Mice. Cell Rep *28*, 896-908 e895.

Celik, M.O., Labuz, D., Keye, J., Glauben, R., and Machelska, H. (2020). IL-4 induces M2 macrophages to produce sustained analgesia via opioids. JCI Insight *5*.

Chen, D., Xie, J., Fiskesund, R., Dong, W., Liang, X., Lv, J., Jin, X., Liu, J., Mo, S., Zhang, T., et al. (2018). Chloroquine modulates antitumor immune response by resetting tumor-associated macrophages toward M1 phenotype. Nat Commun *9*, 873.

Choi, S.Y., Lee, H.H., Lee, J.H., Ye, B.J., Yoo, E.J., Kang, H.J., Jung, G.W., An, S.M., Lee-Kwon, W., Chiong, M., et al. (2016). TonEBP suppresses IL-10-mediated immunomodulation. Sci Rep *6*, 25726.

Dick, S.A., Macklin, J.A., Nejat, S., Momen, A., Clemente-Casares, X., Althagafi, M.G., Chen, J., Kantores, C., Hosseinzadeh, S., Aronoff, L., et al. (2019). Self-renewing resident cardiac macrophages limit adverse remodeling following myocardial infarction. Nat Immunol *20*, 29-39.

Donato, E., Biagioni, F., Bisso, A., Caganova, M., Amati, B., and Campaner, S. (2018). YAP and TAZ are dispensable for physiological and malignant haematopoiesis. Leukemia *32*, 2037-2040.

Dutta, P., and Nahrendorf, M. (2015). Monocytes in myocardial infarction. Arterioscler Thromb Vasc Biol *35*, 1066-1070.

Epelman, S., Lavine, K.J., Beaudin, A.E., Sojka, D.K., Carrero, J.A., Calderon, B., Brija, T., Gautier, E.L., Ivanov, S., Satpathy, A.T., et al. (2014). Embryonic and adult-derived resident cardiac macrophages are maintained through distinct mechanisms at steady state and during inflammation. Immunity *40*, 91-104.

Geng, J., Sun, X., Wang, P., Zhang, S., Wang, X., Wu, H., Hong, L., Xie, C., Li, X., Zhao, H., et al. (2015). Kinases Mst1 and Mst2 positively regulate phagocytic induction of reactive oxygen species and bactericidal activity. Nat Immunol *16*, 1142-1152.

Han, M.S., White, A., Perry, R.J., Camporez, J.P., Hidalgo, J., Shulman, G.I., and Davis, R.J. (2020). Regulation of adipose tissue inflammation by interleukin 6. Proc Natl Acad Sci U S A *117*, 2751-2760.

Heallen, T., Morikawa, Y., Leach, J., Tao, G., Willerson, J.T., Johnson, R.L., and Martin, J.F. (2013). Hippo signaling impedes adult heart regeneration. Development *140*, 4683-4690.

Heidt, T., Courties, G., Dutta, P., Sager, H.B., Sebas, M., Iwamoto, Y., Sun, Y., Da Silva, N., Panizzi, P., van der Laan, A.M., et al. (2014). Differential contribution of monocytes to heart macrophages in steady-state and after myocardial infarction. Circ Res *115*, 284-295.

Horhold, F., Eisel, D., Oswald, M., Kolte, A., Roll, D., Osen, W., Eichmuller, S.B., and Konig, R. (2020). Reprogramming of macrophages employing gene regulatory and metabolic network models. PLoS Comput Biol *16*, e1007657.
Huang, J., Wu, S., Barrera, J., Matthews, K., and Pan, D. (2005). The Hippo signaling pathway coordinately regulates cell proliferation and apoptosis by inactivating Yorkie, the Drosophila Homolog of YAP. Cell *122*, 421-434.
Iyer, S.S., Ghaffari, A.A., and Cheng, G. (2010). Lipopolysaccharide-mediated IL-10 transcriptional regulation requires sequential induction of type I IFNs and IL-27 in macrophages. J Immunol *185*, 6599-6607.
Kim, M.K., Jang, J.W., and Bae, S.C. (2018). DNA binding partners of YAP/TAZ. BMB Rep *51*, 126-133.
Leus, N.G., van der Wouden, P.E., van den Bosch, T., Hooghiemstra, W.T.R., Ourailidou, M.E., Kistemaker, L.E., Bischoff, R., Gosens, R., Haisma, H.J., and Dekker, F.J. (2016). HDAC 3-selective inhibitor RGFP966 demonstrates anti-inflammatory properties in RAW 264.7 macrophages and mouse precision-cut lung slices by attenuating NF-kappaB p65 transcriptional activity. Biochem Pharmacol *108*, 58-74.
Lewandowski, S.L., Janardhan, H.P., and Trivedi, C.M. (2015). Histone Deacetylase 3 Coordinates Deacetylase-independent Epigenetic Silencing of Transforming Growth Factor-beta1 (TGF-beta1) to Orchestrate Second Heart Field Development. J Biol Chem *290*, 27067-27089.
Liu, J., Gao, M., Nipper, M., Deng, J., Sharkey, F.E., Johnson, R.L., Crawford, H.C., Chen, Y., and Wang, P. (2019). Activation of the intrinsic fibroinflammatory program in adult pancreatic acinar cells triggered by Hippo signaling disruption. PLoS Biol *17*, e3000418.
Liu, M., Tong, Z., Ding, C., Luo, F., Wu, S., Wu, C., Albeituni, S., He, L., Hu, X., Tieri, D., et al. (2020). Transcription factor c-Maf is a checkpoint that programs macrophages in lung cancer. J Clin Invest *130*, 2081-2096.
Matsumura, N., Kamei, M., Tsujikawa, M., Suzuki, M., Xie, P., and Nishida, K. (2012). Low-dose lipopolysaccharide pretreatment suppresses choroidal neovascularization via IL-10 induction. PLoS One *7*, e39890.
Matsushita, A., Sato, T., Mukai, S., Fujishita, T., Mishiro-Sato, E., Okuda, M., Aoki, M., Hasegawa, Y., and Sekido, Y. (2019). TAZ activation by Hippo pathway dysregulation induces cytokine gene expression and promotes mesothelial cell transformation. Oncogene *38*, 1966-1978.
Meng, Q., Yang, G., Yang, Y., Ding, F., and Hu, F. (2020). Protective effects of histone deacetylase inhibition by Scriptaid on brain injury in neonatal rat models of cerebral ischemia and hypoxia. Int J Clin Exp Pathol *13*, 179-191.
Mouton, A.J., DeLeon-Pennell, K.Y., Rivera Gonzalez, O.J., Flynn, E.R., Freeman, T.C., Saucerman, J.J., Garrett, M.R., Ma, Y., Harmancey, R., and Lindsey, M.L. (2018). Mapping macrophage polarization over the myocardial infarction time continuum. Basic Res Cardiol *113*, 26.
Nahrendorf, M., Swirski, F.K., Aikawa, E., Stangenberg, L., Wurdinger, T., Figueiredo, J.L., Libby, P., Weissleder, R., and Pittet, M.J. (2007). The healing myocardium sequentially mobilizes two monocyte subsets with divergent and complementary functions. J Exp Med *204*, 3037-3047.
Odashima, M., Usui, S., Takagi, H., Hong, C., Liu, J., Yokota, M., and Sadoshima, J. (2007). Inhibition of endogenous Mst1 prevents apoptosis and cardiac dysfunction without affecting cardiac hypertrophy after myocardial infarction. Circ Res *100*, 1344-1352.
Peng, Y., Chen, B., Zhao, J., Peng, Z., Xu, W., and Yu, G. (2019). Effect of intravenous transplantation of hUCB-MSCs on M1/M2 subtype conversion in monocyte/macrophages of AMI mice. Biomed Pharmacother *111*, 624-630.
Pettersen, J.S., Fuentes-Duculan, J., Suarez-Farinas, M., Pierson, K.C., Pitts-Kiefer, A., Fan, L., Belkin, D.A., Wang, C.Q., Bhuvanendran, S., Johnson-Huang, L.M., et al. (2011). Tumor-associated macrophages in the cutaneous SCC microenvironment are heterogeneously activated. J Invest Dermatol *131*, 1322-1330.

Petty, A.J., Li, A., Wang, X., Dai, R., Heyman, B., Hsu, D., Huang, X., and Yang, Y. (2019). Hedgehog signaling promotes tumor-associated macrophage polarization to suppress intratumoral CD8+ T cell recruitment. J Clin Invest *129*, 5151-5162.
Pinto, A.R., Godwin, J.W., and Rosenthal, N.A. (2014). Macrophages in cardiac homeostasis, injury responses and progenitor cell mobilisation. Stem Cell Res *13*, 705-714.
Pinto, A.R., Paolicelli, R., Salimova, E., Gospocic, J., Slonimsky, E., Bilbao-Cortes, D., Godwin, J.W., and Rosenthal, N.A. (2012). An abundant tissue macrophage population in the adult murine heart with a distinct alternatively-activated macrophage profile. PLoS One *7*, e36814.
Ramjee, V., Li, D., Manderfield, L.J., Liu, F., Engleka, K.A., Aghajanian, H., Rodell, C.B., Lu, W., Ho, V., Wang, T., et al. (2017). Epicardial YAP/TAZ orchestrate an immunosuppressive response following myocardial infarction. J Clin Invest *127*, 899-911.
Rizzo G, V.E., Arampatzi P, Silvestre JS, Zernecke A, Saliba AE, Cochain C. (2020). Single-cell transcriptomic profiling maps monocyte/macrophage transitions after myocardial infarction in mice. bioRxiv.
Ruckerl, D., Jenkins, S.J., Laqtom, N.N., Gallagher, I.J., Sutherland, T.E., Duncan, S., Buck, A.H., and Allen, J.E. (2012). Induction of IL-4Ralpha-dependent microRNAs identifies PI3K/Akt signaling as essential for IL-4-driven murine macrophage proliferation in vivo. Blood *120*, 2307-2316.
Sarode, P., Zheng, X., Giotopoulou, G.A., Weigert, A., Kuenne, C., Gunther, S., Friedrich, A., Gattenlohner, S., Stiewe, T., Brune, B., et al. (2020). Reprogramming of tumor-associated macrophages by targeting beta-catenin/FOSL2/ARID5A signaling: A potential treatment of lung cancer. Sci Adv *6*, eaaz6105.
Shiraishi, M., Shintani, Y., Shintani, Y., Ishida, H., Saba, R., Yamaguchi, A., Adachi, H., Yashiro, K., and Suzuki, K. (2016). Alternatively activated macrophages determine repair of the infarcted adult murine heart. J Clin Invest *126*, 2151-2166.
Song, K., Kwon, H., Han, C., Chen, W., Zhang, J., Ma, W., Dash, S., Gandhi, C.R., and Wu, T. (2020). Yes-Associated Protein in Kupffer Cells Enhances the Production of Proinflammatory Cytokines and Promotes the Development of Nonalcoholic Steatohepatitis. Hepatology *72*, 72-87.
Suelves, N., Kirkham-McCarthy, L., Lahue, R.S., and Gines, S. (2017). A selective inhibitor of histone deacetylase 3 prevents cognitive deficits and suppresses striatal CAG repeat expansions in Huntington's disease mice. Sci Rep *7*, 6082.
Sun, H., Li, D., Yuan, M., Li, Q., Zhen, Q., Li, N., and Wang, G. (2019). Macrophages alternatively activated by endometriosis-exosomes contribute to the development of lesions in mice. Mol Hum Reprod *25*, 5-16.
Sun, X.Y., Qu, Y., Ni, A.R., Wang, G.X., Huang, W.B., Chen, Z.P., Lv, Z.F., Zhang, S., Lindsay, H., Zhao, S., et al. (2017). Novel histone deacetylase inhibitor N25 exerts anti-tumor effects and induces autophagy in human glioma cells by inhibiting HDAC3. Oncotarget *8*, 75232-75242.
Sun, Z., Feng, D., Fang, B., Mullican, S.E., You, S.H., Lim, H.W., Everett, L.J., Nabel, C.S., Li, Y., Selvakumaran, V., et al. (2013). Deacetylase-independent function of HDAC3 in transcription and metabolism requires nuclear receptor corepressor. Mol Cell *52*, 769-782.
Tan, R.Z., Liu, J., Zhang, Y.Y., Wang, H.L., Li, J.C., Liu, Y.H., Zhong, X., Zhang, Y.W., Yan, Y., Lan, H.Y., et al. (2019). Curcumin relieved cisplatin-induced kidney inflammation through inhibiting Mincle-maintained M1 macrophage phenotype. Phytomedicine *52*, 284-294.
Tian, W., Yu, J., Tomchick, D.R., Pan, D., and Luo, X. (2010). Structural and functional analysis of the YAP-binding domain of human TEAD2. Proc Natl Acad Sci U S A *107*, 7293-7298.
Tuladhar, S., Kochanowsky, J.A., Bhaskara, A., Ghotmi, Y., Chandrasekaran, S., and Koshy, A.A. (2019). The ROP16III-dependent early immune response determines the subacute CNS immune response and type III Toxoplasma gondii survival. PLoS Pathog *15*, e1007856.
Ueta, T., Ishihara, K., Notomi, S., Lee, J.J., Maidana, D.E., Efstathiou, N.E., Murakami, Y., Hasegawa, E., Azuma, K., Toyono, T., et al. (2019). RIP1 kinase mediates angiogenesis by modulating macrophages in experimental neovascularization. Proc Natl Acad Sci U S A *116*, 23705-23713.

Vogel, D.Y., Vereyken, E.J., Glim, J.E., Heijnen, P.D., Moeton, M., van der Valk, P., Amor, S., Teunissen, C.E., van Horssen, J., and Dijkstra, C.D. (2013). Macrophages in inflammatory multiple sclerosis lesions have an intermediate activation status. J Neuroinflammation *10*, 35.
Xin, M., Kim, Y., Sutherland, L.B., Murakami, M., Qi, X., McAnally, J., Porrello, E.R., Mahmoud, A.I., Tan, W., Shelton, J.M., et al. (2013). Hippo pathway effector Yap promotes cardiac regeneration. Proc Natl Acad Sci U S A *110*, 13839-13844.
Xin, M., Kim, Y., Sutherland, L.B., Qi, X., McAnally, J., Schwartz, R.J., Richardson, J.A., Bassel-Duby, R., and Olson, E.N. (2011). Regulation of insulin-like growth factor signaling by Yap governs cardiomyocyte proliferation and embryonic heart size. Sci Signal *4*, ra70.
Xu, Q., Choksi, S., Qu, J., Jang, J., Choe, M., Banfi, B., Engelhardt, J.F., and Liu, Z.G. (2016). NADPH Oxidases Are Essential for Macrophage Differentiation. J Biol Chem *291*, 20030-20041.
Yamamoto, S., Yang, G., Zablocki, D., Liu, J., Hong, C., Kim, S.J., Soler, S., Odashima, M., Thaisz, J., Yehia, G., et al. (2003). Activation of Mst1 causes dilated cardiomyopathy by stimulating apoptosis without compensatory ventricular myocyte hypertrophy. J Clin Invest *111*, 1463-1474.
Yu, T., Gan, S., Zhu, Q., Dai, D., Li, N., Wang, H., Chen, X., Hou, D., Wang, Y., Pan, Q., et al. (2019). Modulation of M2 macrophage polarization by the crosstalk between Stat6 and Trim24. Nat Commun *10*, 4353.
Zhang, J., Muri, J., Fitzgerald, G., Gorski, T., Gianni-Barrera, R., Masschelein, E., D'Hulst, G., Gilardoni, P., Turiel, G., Fan, Z., et al. (2020a). Endothelial Lactate Controls Muscle Regeneration from Ischemia by Inducing M2-like Macrophage Polarization. Cell Metab *31*, 1136-1153 e1137.
Zhang, Y., Li, X., Luo, Z., Ma, L., Zhu, S., Wang, Z., Wen, J., Cheng, S., Gu, W., Lian, Q., et al. (2020b). ECM1 is an essential factor for the determination of M1 macrophage polarization in IBD in response to LPS stimulation. Proc Natl Acad Sci U S A *117*, 3083-3092.
Zhao, C., Mirando, A.C., Sove, R.J., Medeiros, T.X., Annex, B.H., and Popel, A.S. (2019). A mechanistic integrative computational model of macrophage polarization: Implications in human pathophysiology. PLoS Comput Biol *15*, e1007468.
Zhou, X., Li, W., Wang, S., Zhang, P., Wang, Q., Xiao, J., Zhang, C., Zheng, X., Xu, X., Xue, S., et al. (2019). YAP Aggravates Inflammatory Bowel Disease by Regulating M1/M2 Macrophage Polarization and Gut Microbial Homeostasis. Cell Rep *27*, 1176-1189 e1175.
