# Supplementary material for: YAP/TAZ deficiency reprograms macrophage phenotype and improves infarct healing and cardiac function after myocardial infarction
Source: PLoS Biol. 2020 Dec 2;18(12):e3000941. doi: 10.1371/journal.pbio.3000941 (PMC7735680; doi:10.1371/journal.pbio.3000941)
Supplement: Attachment — Submitted filename: Response to Reviewers.pdf [file pbio.3000941.s046.pdf]

Reviewer #1:

Comment: I have no further questions. The authors have adequately addressed my concerns. I do believe that it is more appropriate to use the terms "pro-inflammatory" and "reparative" phenotypes as opposed to M1/M2.

Response: *We would like to thank the reviewer for his/her positive evaluation of our manuscript. We have replaced the term M1/M2 with "pro-inflammatory" and "reparative" phenotypes in the revised manuscript.*

Reviewer #2:

Comment: I have no further comments. The authors adequately addressed my concerns.

Response: *We would like to thank the reviewer for his/her positive evaluation of our manuscript.*

Reviewer #3:

Comment: The Authors have made a significant effort to address the comments raised by all of the reviewers. I am satisfied by the responses of the authors and by the additional experiments included. I particularly appreciate the experiments to investigate the enzymatic vs structural role of HDAC3.

Response: *We would like to thank the reviewer for his/her positive evaluation of our manuscript.*

Minor comments:

Comment: in Figure 6 D, the y axis is labelled Collagen 1 Production. The legend indicates that this is collagen 1 +ve area of the section. The label collagen 1 production is misleading. Please replace with Collagen 1 +ve area.

Response: *We have changed Collagen 1 Production to Collagen 1^{+} area in both Figure 6D and 7J of the revised manuscript.*

Comment: Could the authors indicate whether their Echo is 3D. If only 2D echo, all volumes are based based on 2 D dimensions that as one of the other referees indicated may not accurately reflect alterations due to MI - which may be asymmetric in nature.

Response: *This information is provided in the "Materials and Methods" section of the manuscript. All the data are from 2D echo which is the most commonly used method for evaluating cardiac function after MI. To evaluate the cardiac function efficiently, an average of 10 cardiac cycles of standard 2 dimensional (2D) parasternal long and short axis (mid papillary muscle level), as well as pulsed-wave Doppler recording of the mitral inflow, were acquired and stored for subsequent offline analysis.*

We agree with Reviewer 3 that Reviewer 1 suggested that "Given the asymmetric pattern of injury and left ventricular (LV) remodeling, fractional shortening is not an ideal measurement. The authors should include quantitative data pertaining to LV ejection fraction, LV diastolic and systolic volumes, and LV mass". To address this point, We have included additional quantitative data on LV ejection fraction, End Systolic Volume (LVESV), End Diastolic Volume (LVEDV), Stroke Volume (SV), LV mass, and Fractional Area Change (FAC) (Figure 5 and Supplementary Figure 14) in the revised manuscript.

Comment: Page 28 (page 4 of MS), Para 2, line 21. in the sentence: development but also for limiting the inflammatory and fibrotic response during post-MI recovery phage. The last word should be phase.

Response: *We have corrected the text in the revised manuscript.*

Comment: Page 38 (page 10 in MS), Para 1, line 9. in the sentence : Alternatively, a decreased in the expression of.... decreased should be replaced with decrease.

Response: *We have corrected the text in the revised manuscript.*
